# Chemically augmented malaria sporozoites display an altered immunogenic profile

**DOI:** 10.3389/fimmu.2023.1204606

**Published:** 2023-08-31

**Authors:** Nikolas Duszenko, Roos van Schuijlenburg, Severine Chevalley-Maurel, Danny M. van Willigen, Laura de Bes-Roeleveld, Stefanie van der Wees, Chanel Naar, Els Baalbergen, Graham Heieis, Anton Bunschoten, Aldrik H. Velders, Blandine Franke-Fayard, Fijs W. B. van Leeuwen, Meta Roestenberg

**Affiliations:** ^1^ Department of Parasitology, Leiden University Medical Center, Leiden, Netherlands; ^2^ Interventional Molecular Imaging Laboratory, Department of Radiology, Leiden University Medical Center, Leiden, Netherlands; ^3^ Laboratory of BioNanoTechnology, Wageningen University & Research, Wageningen, Netherlands

**Keywords:** malaria, vaccine, adjuvant, immunogenicity, supramolecular chemistry

## Abstract

Despite promising results in malaria-naïve individuals, whole sporozoite (SPZ) vaccine efficacy in malaria-endemic settings has been suboptimal. Vaccine hypo-responsiveness due to previous malaria exposure has been posited as responsible, indicating the need for SPZ vaccines of increased immunogenicity. To this end, we here demonstrate a proof-of-concept for altering SPZ immunogenicity, where supramolecular chemistry enables chemical augmentation of the parasite surface with a TLR7 agonist-based adjuvant (SPZ-SAS(CL307)). *In vitro*, SPZ-SAS(CL307) remained well recognized by immune cells and induced a 35-fold increase in the production of pro-inflammatory IL-6 (p < 0.001). More promisingly, immunization of mice with SPZ-SAS(CL307) yielded improved SPZ-specific IFN-γ production in liver-derived NK cells (percentage IFN-γ^+^ cells 11.1 ± 1.8 vs. 9.4 ± 1.5%, p < 0.05), CD4^+^ T cells (4.7 ± 4.3 vs. 1.8 ± 0.7%, p < 0.05) and CD8^+^ T cells (3.6 ± 1.4 vs. 2.5 ± 0.9%, p < 0.05). These findings demonstrate the potential of using chemical augmentation strategies to enhance the immunogenicity of SPZ-based malaria vaccines.

## Graphical Abstract

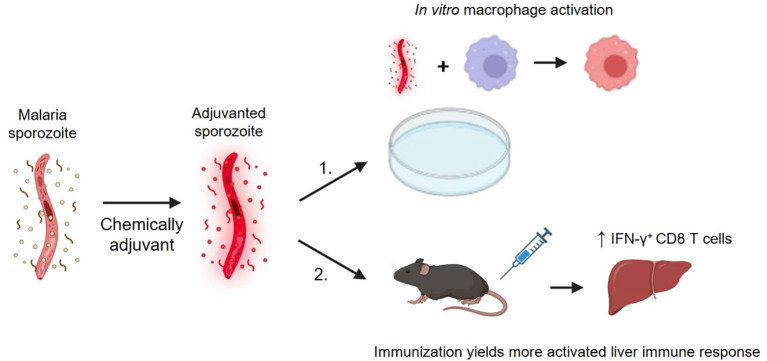

## Introduction

The World Health Organization’s recent endorsement of the world’s first malaria vaccine, RTS,S, marks an important step in the fight against malaria. RTS,S – a subunit-based vaccine that targets the circumsporozoite protein (CSP) abundantly expressed on malaria sporozoites (SPZ) – has yielded a protective efficacy of ±30% (1). To improve on these numbers, whole-cell vaccines based on SPZ offer a promising alternative, as they display a broader range of antigens and can induce high levels of protection in malaria-naïve populations (2–4). Such protection appears primarily mediated by cytotoxic cellular immune responses, for example in an increased proportion of IFN-γ^+^ CD8 T cells and NK cells (5–7). Problematically, however, this high efficacy in malaria-naïve populations has thus far not been replicated in malaria-endemic settings using similar vaccination regimens (8, 9). It thus appears that in individuals pre-exposed to malaria, the intrinsic tolerogenicity of SPZ blunts the induction of robust immune responses to SPZ vaccines, though the mechanisms hereof remain incompletely understood (10–12).

A well-established approach to overcoming suboptimal vaccine immunogenicity is supplementing the vaccine with adjuvants, immunogenic compounds that induce (pro-inflammatory) activation (13). Though such strategies are nowadays routinely applied for subunit vaccines, their use for whole microbe-based vaccines is an untested concept. Adjuvanted subunit vaccines, as well as next-generation nanoparticle vaccines, have been shown to be most potent when vaccine and adjuvant are somehow physically linked to ensure co-delivery to immune cells (14–17). Devising an analogous vaccine formulation that co-delivers microbe and adjuvant, while conceptually challenging, may thus be important for improving SPZ-based vaccines’ immunogenicity. Furthermore, given the preferred intravenous administration of SPZ-based vaccines, accurate co-delivery of vaccine and adjuvant is critical to promoting targeted (antigen-specific) immune activation whilst minimizing harmful systemic effects.

Chemically mediated cell surface functionalization offers a potential route to realizing SPZ/adjuvant co-delivery. Numerous strategies have been reported showing the feasibility of harnessing diverse chemical methodologies to functionalize cell surfaces (18). In our group, we have developed one such strategy, wherein supramolecular host-guest chemistry is leveraged to functionalize microparticles (19), eukaryotic cells (20) and bacteria (21). More recently, we have shown that supramolecular functionalization of bacteria with an adjuvant yields improved immunogenicity (22). Based on this work, we reasoned that it should similarly be possible to chemically augment the malaria SPZ cell surface with adjuvants, and in doing so alter the parasite’s immunogenicity.

Here, we demonstrate the conceptual feasibility of chemically augmenting *Plasmodium berghei* SPZ (PbSPZ) with a so-called “**S**upramolecular **A**djuvanting **S**ystem” (SAS) that introduces a TLR7 agonist, CL307, onto the SPZ surface – a type of adjuvant known to induce the sort of cytotoxic cellular immune responses seemingly important for anti-malarial protection (23–25). These chemically augmented PbSPZ, referred to as PbSPZ-SAS(CL307), had their immunogenicity assayed *in vitro* via macrophage model and *in vivo* using a mouse immunization model.

## Methods

### Chemical synthesis/analysis miscellany

Chemicals were obtained commercially from Merck (Darmstadt, Germany), TCI (Tokyo, Japan), or Cyclodextrin-Shop (Tilburg, The Netherlands) and used without further purification. Amino acids were obtained from either Bachem (Bubendorf, Switzerland) or Iris Biotech (Marktredwitz, Germany). Solvents were obtained from Actu-All (Oss, The Netherlands), Biosolve (Valkenswaard, The Netherlands), or Merck (Darmstadt, Germany). Acetonitrile, N,N-dimethylformamide, and dimethylsulfoxide were dried using 4Å molecular sieves from Merck (Darmstadt, Germany) unless stated otherwise. Reactions were carried out under normal atmosphere unless stated otherwise. Column chromatography was performed with 40–63 µm silica from Screening Devices (Amersfoort, The Netherlands). SPPS was carried out either by a Biotage Syro II (Uppsala, Sweden) or by hand using fritted tubes (6, 10, or 25 mL) from Screening Devices (Amersfoort, The Netherlands) and in-house N_2_ flow/vacuum.

High-performance liquid chromatography was performed on a Waters HPLC system using either a 1525EF or 2545 pump and a 2489 UV/VIS detector. For preparative HPLC either a Dr. Maisch GmbH Reprosil-Pur 120 C18-AQ 10 μm (250 × 20 mm) column using a flow of 12 mL/min or an XBridge Prep C8 10 μm OBD (250 × 30 mm column) with a flow of 25 mL/min was used. For semi-preparative HPLC, a Dr. Maisch GmbH Reprosil-Pur C18-AQ 10 μm (250 × 10 mm) column was used with a flow of 5 mL/min. For analytical HPLC a Dr. Maisch GmbH Reprosil-Pur C18-AQ 5 μm (250 × 4.6 mm) column with a flow of 1 mL/min and a gradient of 5 ➔ 95% CH_3_CN in H_2_O) (0.1% TFA) in 40 min (1 mL/min) was used. Mass spectrometry was performed using a Bruker Microflex Matrix-assisted laser desorption ionization time-of-flight (MALDI-TOF) mass spectrometer (Billerica, MA, United States). ^1^H NMR, COSY, and ^13^C NMR of the dyes were recorded on a Bruker AV-300 spectrometer (300 MHz) (Billerica, MA, United States) in methanol-d_4_. The quantification of the number of β-CD units per polymer with ^1^H NMR and DOSY was done in D_2_O using a Bruker Avance III spectrometer (500 MHz), equipped with a 5 mm TXI probe. Dialysis was performed using Pur-A-Lyzer (either Mega/Maxi 3500 MWCO or Mini 12000 MWCO) dialysis kits from (Sigma-Aldrich, St. Louis, MO, USA). Absorbance spectra were recorded using an Ultrospec 2100 pro (Amersham Biosciences, Little Chalfont, United Kingdom). The analyses can be found in the **Supplementary Material**.

### Synthesis of chemical compounds

Ad-Osu esters were prepared as follows: amineC3Ad-(SO3)Cy3(SO3)COOH was dissolved in 330μL of dried DMSO. DiPEA (1uL, 5.83μmol) and 20uL of a 10mg/mL HsPyU were added and the reaction mixture was left to stir at room temperature for 90 minutes. Subsequently, MilliQ and CH_3_CN were added to the solution and the crude product was purified using preparative HPLC (15 - 95% CH_3_CN in Milli Q over 46 minutes) to yield the title product as a blue solid. This solid was resuspended in DMSO to yield a 1.2 mM stock for downstream applications.

The CL307-bearing host polymers were synthesized based on a previously published procedure (20). To initial graft β-cyclodextrins onto the polymers, poly(isobutylene-alt-maleic-anhydride (200.0 mg, 3.3 µmol) was dissolved in dimethylsulfoxide (3.0 mL), whereafter amino(6-monodeoxy-6-mono)-β-cyclodextrin hydrochloride (620.3 mg, 530.0 µmol) and N,N-diisopropylethylamine (29.0 µL, 166.7 µmol) were added, and stirring at 80°C was carried out for 94 hours. The solution was purified by dialysis in water (1000.0 mL) for 7 hours, followed by dialysis in phosphate buffer (0.2 M, pH 9, 1000.0 mL) for 144 hours including refreshment of buffer twice, followed by dialysis in water (1000.0 mL) for 7 hours. The dialysate was discarded and the residue was lyophilized, yielding an off-white solid (453.6 mg, 85.1% isolated yield).

Next, Cy5 dyes, used to track the construct throughout experiments, were coupled using a method published by Fattahi et al. (26). PIBMA_[389]_-CD_[85]_ (420.0 mg, 2.6 µmol) was dissolved in water, whereafter N,N’-diisopropylcarbodiimide (122.8 µL, 798.0 µmol) was added. The mixture was stirred at room temperature for 1 hour followed by the addition of 2.4 mL of a 1.1 mg/mL solution of NH_2_-Cy5-COOH (5) in 1:8 ethanol/water (2.8 mg, 5.3 µmol). The solution was stirred for 5 hours at room temperature whereafter it was dialyzed in water (5000.0 mL) for 24 hours while refreshing the water once, followed by lyophilization of the residue.

Thereafter, CL307 (Invitrogen, Waltham, MA, USA) was coupled to polymers by dissolving PIBMA_[389]_-CD_[85]_-Cy5_[2_ **_]_** (8.05 mg, 50.3 nmol) in water (805 µL), followed by addition of DIC (7.7 µL, 50.3 µmol). After stirring for 1.3 hours at room temperature, CL307 (1.5 mg, 2.5 µmol) in water (1.5 mL) was added. After shaking for 1.3 hours ethanolamine (9.1 µL, 150.9 µmol) was added (to react with leftover free carboxylates and thereby sequester the polymers’ negative charges), and stirring was continued for another 16 hours at room temperature. Thereafter, the reaction mixture was dialyzed in water (5 L) for 29 hours with one refreshment of water. The residue was used as is for experiments; PBS pH 7.4 was added where necessary.

### Production of PbSPZ

*Plasmodium berghei* SPZ (PbSPZ) were produced in *Anopheles* mosquitoes that fed on male OF1 mice 4-5 days prior infected with *P. berghei* and having reached a blood gametocytemia of 0.2-0.8%.

### Supramolecular complexation of CL307-bearing host polymers (SAS(CL307)) onto PbSPZ to generate PbSPZ-SAS(CL307)

Salivary glands from infected mosquitoes containing PbSPZ, and from uninfected mosquitoes for salivary gland extra (SGE) controls, were manually dissected at days 21-28 post-infection. Immediately prior to use, glands were homogenized to isolate PbSPZ. Isolated PbSPZ were pre-functionalized by incubating in 100 uL RPMI (Gibco, Thermo Fisher, Waltham, MA, USA) containing 1 µM Ad-OSu ester, a construct reactive toward the cell surface lysine residues present on the circumsporozoite protein sheath enveloping the parasite, and 10 µg/mL Hoechst 33342 for 15 minutes at 37°C. Thereafter, PbSPZ were twice washed in 1.3 mL PBS supplemented with 1% fetal bovine serum (FBS) by centrifugation at 13k RCF for 5 minutes and decanting of supernatant. PbSPZ were resuspended in the remaining supernatant by pipetting and bringing the total volume to 100 µL PBS, followed by the addition of 100 µL of 2 µM CL307-bearing host polymer (CD_85_Cy5_2_CL307_57_PIBMA_389_) in 1.9X PBS (where 1X is standard PBS concentration) and incubation for 4 hours at 37°C that included mixing by pipet every 30 minutes. Then, PbSPZ were thrice washed in 1.3 mL PBS supplemented with 1% FBS by centrifugation at 13k RCF for 5 minutes and decanting of supernatant. PbSPZ were resuspended by pipetting and counted via Buerker chamber, and then ready for downstream applications.

### Confocal imaging of PbSPZ-SAS(CL307)

PbSPZ-SAS(CL307) were added to glass-bottom confocal dishes (MatTek, Ashland, MA, USA) at a density of 25k in 10 µL and overlaid with a coverslip. Confocal dishes were sealed with parafilm and left for 30 minutes in a humidified chamber to allow parasites to settle to the bottom. Imaging of PbSPZ-SAS(CL307) was performed on a Leica (Wetzlar, Germany) SP8 confocal fluorescence microscope.

### Flow cytometric analysis of PbSPZ-SAS(CL307)

PbSPZ-SAS(CL307) at a density of 100k/mL were run through a BD (Franklin Lakes, NJ, USA) FACSCantoII instrument. Gating on the Hoechst signal discriminated PbSPZ-SAS(CL307) from contaminating salivary gland debris. Cy5 signal originating from the complexed CL307-bearing host polymers was measured in the instrument’s “APC” channel. Post-acquisition analysis was performed using FlowJo 10 (Ashland, OR, USA).

### Production of bone marrow-derived macrophages

Femur and tibia bones from female C57BL/6 mice were washed for 1 minute in ethanol and then rinsed with RPMI. Bones’ ends were cut, and the marrow within was flushed out via a syringe into RPMI. Cells were centrifuged at 300 RCF for 10 minutes at 4°C, the supernatant was discarded by decant, and cells were resuspended in 3 mL red blood cell lysis buffer (specs) for 3 minutes on ice. Cells had added to them 7 mL RPMI, centrifuged again at 300 RCF for 10 minutes at 4°C, supernatant discarded, and resuspended in 5 mL TCM (RPMI + 5% FBS + 0.1% β-mercaptoethanol). Cells were counted by Buerker counting chamber and subsequently plated in plastic Petri dishes (Thermo Fisher) at a density of 2*10^7^ cells in 10 mL TCM supplemented with 20 ng/mL murine M-CSF (derived in-house via M-CSF-producing L929 cells) to promote differentiation of monocytes into macrophages. The medium was refreshed once on day 2. On day 6, the medium was removed from cells, cells rinsed once with PBS, and then incubated with 2 mL Accutase (STEMCELL Technologies, Vancouver, Canada) for 15 minutes at 37°C to promote macrophage detachment. Eight mL of TCM was added and macrophages were resuspended by serological pipet. Macrophages were centrifuged at 300 RCF for 10 minutes at 4°C and the supernatant was discarded, followed by resuspension of cell pellets in 5 mL TCM by serological pipet. Macrophages were counted by Buerker counting chamber and then ready for downstream applications.

### Confocal imaging of macrophage phagocytosis of PbSPZ-SAS(CL307)

Harvested macrophages were plated at a density of 200k in 200 µL TCM onto glass-bottom confocal dishes (MatTek) and placed overnight into a 37°C incubator to allow macrophages to adhere. The next day, 1.8 mL TCM containing 10 µg/mL Hoechst 33342 was added per dish prior to imaging. Confocal dishes were positioned on an Andor (Belfast, UK) Dragonfly 500 spinning disk fluorescence microscope and prepared for image acquisition. Immediately prior to acquisition, 50k PbSPZ-SAS(CL307) in 10 µL were carefully added just above the macrophage layer. Images were acquired as z-stacks of approximately 40 µm in depth at an interval of 1 µm every 2.5 minutes for a total of 30 minutes. Post-acquisition analysis was performed using Imaris software (Zurich, Switzerland).

### Flow cytometry quantitation of macrophage phagocytosis of PbSPZ-SAS(CL307)

Harvested macrophages were plated at a density of 100k in 100 µL TCM into flat-bottom 96-well plates (vendor) and placed overnight into a 37°C incubator to allow macrophages to adhere. The next day, 100 µL aliquots of 50k, 25k or 12.5k PbSPZ-SAS(CL307) or wild-type SPZ tagged with a FITC-based mitotracker (Thermo Fisher) were added per well, and the plate centrifuged at 300 RCF for 10 minutes at room temperature. Plates were incubated for 45 minutes at 37°C. Then, the supernatant was removed by pipet, and 200 µL cold FACS buffer (PBS supplemented with 0.5% bovine serum albumin and 20 mM EDTA) was added per well. Plates were incubated on ice for 15 minutes to promote macrophage detachment. Complete detachment was achieved by manual scraping with pipet tips and resuspending by pipet, after which macrophages were transferred to FACS tubes. Macrophages were finally run through a BD FACSCanto II instrument to assess the presence of FITC-tagged SPZ within. Post-acquisition analysis was performed using FlowJo 10 software.

### *In vitro* immune response of macrophages to PbSPZ-SAS(CL307)

Harvested macrophages were plated at a density of 100k in 100 µL TCM into flat-bottom 96-well plates (Thermo Fisher) and placed overnight into a 37°C incubator to allow macrophages to adhere. The next day, 100 µL aliquots of 50k PbSPZ-SAS(CL307) or relevant controls were added per well, and the plates were incubated at 37°C for 24 hours. Thereafter, cell culture supernatants were removed and stored at -80°C for eventual cytokine analysis. 200 µL cold PBS was added per well, and the plates were incubated for 15 minutes on ice to promote macrophage detachment. Complete detachment of macrophages was achieved by manual scraping with pipet tips and resuspending by pipet, after which macrophages were transferred to V-bottom 96-well plates (Thermo Fisher) on ice. Macrophages were centrifuged at 200 RCF for 4 minutes at 4°C and the supernatant was discarded by decant. Cell pellets were resuspended in 50 µL 400-fold diluted (in PBS) Aqua live/dead stain (Thermo Fisher) and incubated for 20 minutes on ice in the dark. Then, 150 µL 1.9% paraformaldehyde (Thermo Fisher) in PBS was added per well to fix cells, followed by another 15 minutes of incubation on ice in the dark. Macrophages were then centrifuged at 200 RCF for 4 minutes at 4°C and the supernatant was discarded by decant. Cell pellets were resuspended in 200 µL FACS buffer by pipet and centrifuged at 200 RCF for 4 minutes at 4°C, followed by supernatant decant. Cell pellets were finally resuspended in 200 µL FACS buffer and macrophages were stored at 4°C for up to a week prior to FACS analysis. On the day of FACS analysis, macrophages were centrifuged at 200 RCF for 4 minutes at 4°C, followed by supernatant decant. Cell pellets were resuspended in 30 µL of antibody mix (see **Table 1**) and incubated for 30 minutes at 4°C in the dark. Thereafter, 170 µL of FACS buffer was added, macrophages were centrifuged at 200 RCF for 4 minutes at 4°C, and the supernatant was discarded by decant. Cell pellets were resuspended in 80 µL FACS buffer and transferred to FACS tubes. Macrophages were finally run through a BD Fortessa flow cytometer to measure surface marker expression levels. Post-acquisition analysis was performed using FlowJo 10 software.

**Table 1:** Antibody cocktail used to assay *in vitro* macrophage activation in response to PbSPZ-SAS(CL307) stimulation via flow cytometry.

Target	Antibody clone	Fluorochrome
CD11b	M1/70	PE-Cy7
CD80	RM80	PE
CD86	GL-1	BV421
CD206	C068C2	FITC
CD274	10F-9G2	BV711
F4/80	BM8	APC

### Mouse model for immunization studies

Female C57BL/6J mice (Charles River Laboratories, France) were obtained at 4-6 weeks of age and acclimatized for 1 week prior to use. Mice were group-housed (random allocation to groups of same-sex littermates) in ventilated cages with autoclaved aspen woodchip, fun tunnel, wood chew block and nestlets (12:12 hour (h) light-dark cycle; 21 ± 2°C; relative humidity of 55 ± 10%), and fed with a commercially-prepared, autoclaved dry rodent diet pellets and provided with water, both available *ad libitum*. Animal experiments were granted with a license AVD1160020173304 by the Competent Authority after advice on ethical evaluation by the Animal Experiments Committee Leiden, and were performed in accordance with the Experiments on Animals Act (Wod, 2014), the applicable legislation in the Netherlands in accordance with the European guidelines (EU directive no. 2010/63/EU). Experiments were executed in a licensed establishment for experimental animals.

### *In vivo* immunization of mice with PbSPZ-SAS(CL307)

Mice were immunized with 25k PbSPZ-SAS(CL307) or wild-type SPZ in 200 µL PBS by injection into the tail vein, with negative controls receiving the PBS vehicle only. The initial prime immunization took place on day 0, with a second boost following on day 7.

### Processing of tissues (liver, spleen, blood) from immunized mice

Fourteen days after the initial prime immunization, mice were anesthetized by a 10% ketamine (Dechra Pharmaceuticals, Northwich, UK)/20 mg/mL xylazine (Alfasan, Woerden, The Netherlands) cocktail. Following this, animals were perfused with 10 mL PBS, followed by excision of the liver and spleen. These organs were immediately processed as follows.

To process livers, livers were minced by scalpel and added to 20 mL RPMI containing 1 mg/mL Collagenase IV (Sigma-Aldrich, St. Louis, MO, USA) and 2000 U/mL DNase I (Sigma-Aldrich) in 50 mL conical tubes. Livers were digested for 45 minutes at 37°C, with one mix of tubes’ contents by inversion halfway through. Thereafter, digested livers were run through 100 µm filters (BD), and filters were washed twice with 10 mL aliquots of Wash Buffer (WB – PBS supplemented with 1% FBS and 2.5 mM EDTA). Tubes were spun at 50 RCF for 3 minutes at 4°C to pellet hepatocytes. Supernatants were carefully removed by serological pipet, leaving behind the hepatocyte cell pellets, and transferred to fresh 50 mL tubes. Hepatocyte-bereft cells were washed once by centrifugation at 300 RCF for 10 minutes at 4°C and careful decant of supernatant, resuspension in 20 mL WB, and identical centrifugation/decant. Cell pellets were resuspended in 3 mL red blood cell lysis buffer and incubated on ice for 2 minutes, followed by the addition of 7 mL WB. Cells were centrifuged at 300 RCF for 10 minutes at 4°C and the supernatant was carefully discarded by decant. Resuspension in 10 mL MACS buffer (PBS with 0.5% bovine serum albumin and 20 mM EDTA) was followed by identical centrifugation/decant. Cell pellets were resuspended in 1 mL MACS buffer and 35 µL CD45 MicroBeads (Miltenyi Biotec, Bergisch Gladbach, Germany) was added to them. After 15 minutes of incubation at 4°C, cells had added to them 10 mL MACS buffer and were centrifuged at 300 RCF for 10 minutes at 4°C, followed by careful decant of supernatant. Cell pellets were resuspended in 5 mL MACS buffer and run through pre-wetted LS columns (Miltenyi Biotec) attached to a magnetic stand. These columns were thrice washed with 3 mL MACS buffer afterward. Columns were removed from magnetic stands and CD45^+^ cells therein displaced by flushing the column vigorously with 5 mL RPMI, which was thereafter supplemented with 1% FBS. CD45^+^ cells, i.e. liver leukocytes, were centrifuged at 300 RCF for 10 minutes at 4°C, had supernatant decanted, and were finally resuspended in 2 mL TCM. (Viable) cells were counted using a Buerker counting chamber and trypan blue, and then ready for downstream applications.

To process spleens, spleens in 1 mL HBSS (Gico) in 24-well flat-bottom plates (Thermo Fisher) were first mashed with a syringe plunger and then digested with 1 mg/mL Collagenase D (Roche, Basel, Switzerland) and 2000 U/mL DNase I (Sigma-Aldrich) for 20 minutes at 37°C. Digestate was then passed through 100 µm filters that were thrice rinsed with 3 mL MACS buffer. Filtered cells were centrifuged at 300 RCF for 10 minutes at 4°C and the supernatant was discarded. Cell pellets were resuspended in 3 mL red blood cell lysis buffer and incubated for 2 minutes on ice, followed by the addition of 7 mL MACS buffer. Cells were centrifuged at 300 RCF for 10 minutes at 4°C and the supernatant was discarded by decant. Cell pellets were resuspended in 10 mL TCM. (Viable) cells were counted using a Buerker counting chamber and trypan blue, and then ready for downstream applications.

### Analysis of liver and spleen bulk immunophenotypes

To gauge general liver and spleen immunoreactivity post immunization independent of (malaria) specific stimulus, we measured cytokine secretion of these cells into culture supernatants over the course of 36 hours after receiving a generic activation stimulus of phorbol 12-myristate 13-acetate (PMA) and ionomycin. For this, liver leukocytes and splenocytes were plated at a density of 200k and 500k per well, respectively, into U-bottom 96-well plates (Thermo Fisher) in 100 µL TCM. Each well then received an additional 100 µL of TCM containing PMA at a concentration of 0.2 µg/mL and ionomycin at a concentration of 2 µg/mL. Cells were then incubated for 36 hours at 37°C. Thereafter, cell culture supernatants were removed and stored at -20°C for eventual cytokine analysis by cytometric bead assay (BD) according to the manufacturer’s specifications.

### Analysis of liver cellular immune responses

Liver leukocytes were plated at a density of 200k per well into U-bottom 96-well plates (Thermo Fisher) in 100 µL TCM. Each well then received an additional 100 µL of TCM containing 100k SPZ and 20 µg/mL brefeldin A. Cells were then incubated for 4 hours at 37°C. Thereafter, liver leukocytes were transferred to V-bottom 96-well plates (Thermo Fisher) on ice. Liver leukocytes were centrifuged at 200 RCF for 4 minutes at 4°C and the supernatant was discarded by decant. Cell pellets were resuspended in 50 µL 400-fold diluted (in PBS) Aqua live/dead stain (Thermo Fisher) and incubated for 20 minutes on ice in the dark. Then, 150 µL 1.9% paraformaldehyde in PBS was added per well to fix cells, followed by another 15 minutes of incubation on ice in the dark. Cells were then centrifuged at 200 RCF for 4 minutes at 4°C and the supernatant was discarded by decant. Cell pellets were resuspended in 200 µL FACS buffer by pipet and centrifuged at 200 RCF for 4 minutes at 4°C, followed by supernatant decant. Cell pellets were finally resuspended in 200 µL FACS buffer and liver leukocytes were stored at 4°C for up to 2 days prior to FACS analysis. On the day of FACS analysis, liver leukocytes were centrifuged at 200 RCF for 4 minutes at 4°C, followed by supernatant decant. Cell pellets were resuspended in 30 µL of antibody mix (see **Table 2**) and incubated for 45 minutes at 4°C in the dark. Thereafter, 170 µL of FACS buffer was added, liver leukocytes were centrifuged at 200 RCF for 4 minutes at 4°C, and the supernatant was discarded by decant. Cell pellets were resuspended in 80 µL FACS buffer and transferred to FACS tubes. Liver leukocytes were finally run through a BD Fortessa flow cytometer to measure surface marker expression levels. Post-acquisition analysis was performed using FlowJo 10 software.

Table 2Antibody cocktails used to assay *in vivo* immune cell activation in livers and spleens of mice immunized with PbSPZ-SAS(CL307).

**Table:** 

Panel 1 (T cell and NK cell responses)		
Target	Antibody clone	Fluorochrome
CD4	RM4-5	BV650
CD8	53-6.7	BV711
CD44	IM7	AF700
CD45	30-F11	PerCP-Cy5.5
CD69	H1.2F3	PE-CF594
CTLA-4	UC10-4B9	PE
CXCR3	CXCR3-173	BV421
γδ-TCR	GL3	BV605
IFN-γ	XMG1.2	FITC
IL-2	JES6-1A12	PE-Cy5
KLRG1	2F1/KLRG1	APC
NK-1.1	PK136	APC-Cy7
PD-1	RMP1-30	BV786
TNF-α	MP6-XT22	PE-Cy7

**Table:** 

Panel 2 (myeloid and B cell responses)		
Target	Antibody clone	Fluorochrome
B220	RA3-6B2	PE-Cy5
CD3	17A2	PE-Dz594
CD11b	M1/70	APC-eF780
CD11c	N418	FITC
CD25	PC61	PerCP-Cy5.5
CD27	LG.3A10	BV605
CD45	30-F11	BV785
CD86	GL-1	PE
F4/80	BM-8	BV711
IL-12	C15.6	APC
Ly6C	HK1.4	BV650
MHCII	M5/114.15.2	AF700
PD-L1	B7-H1	PE-Cy7
XCR1	ZET	BV421

### Statistical analyses

Statistical analyses were performed using IBM SPSS Statistics 25 and Graphpad Prism 9.3.1 software. Details thereof can be found in the article’s figure legends. Significance was defined as a p-value of less than 0.05. Sample size estimations where appropriate were performed with a power analysis using an alpha of 0.05 and a power of 90%. Data subjected to parametric statistical analyses had its normality confirmed beforehand.

## Results

### Chemical augmentation of PbSPZ yields adjuvanted PbSPZ-SAS(CL307)

**Figure 1:**
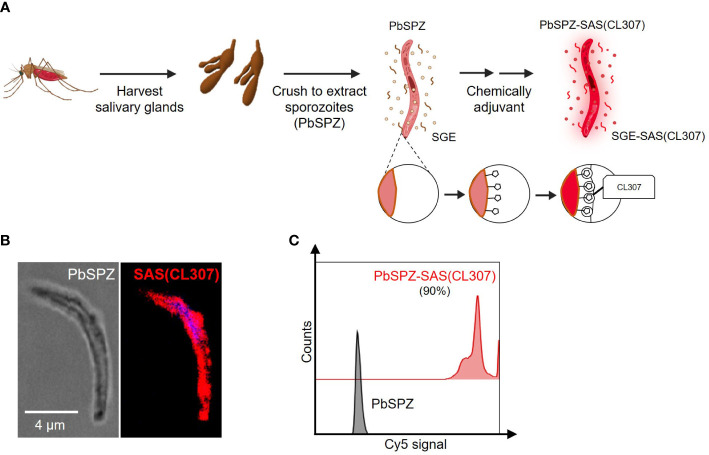
Chemical augmentation of PbSPZ yields adjuvanted PbSPZ-SAS(CL307). **(A)** Schematic illustrating the generation of chemically augmented PbSPZ-SAS(CL307). Malaria-infected mosquitoes were dissected to harvest PbSPZ-containing salivary glands. Pooled salivary glands were crushed to obtain PbSPZ in salivary gland extract (SGE), which were then chemically augmented with adjuvant by complexing onto their surface CL307-bearing supramolecular polymers (SAS(CL307)) to yield PbSPZ-SAS(CL307). **(B)** Confocal fluorescence microscopy image of a PbSPZ-SAS(CL307) (left: brightfield) with SAS(CL307) (right: Cy5 signal) complexed to the surface. **(C)** Flow cytometry histograms of Cy5 signal of PbSPZ-SAS(CL307) (top: red) and wild-type SPZ (bottom: grey). PbSPZ, *P. berghei* sporozoite; SAS, supramolecular adjuvanting system; CL307, a Toll-like receptor 7 agonist; SGE, salivary gland extract.

To generate chemically augmented PbSPZ-SAS(CL307), PbSPZ in salivary gland extract (SGE) were harvested from infected mosquitoes and pre-functionalized with adamantane (the supramolecular guest), followed by host-guest complexation with poly(isobutylene-alt-maleic anhydride) (PIBMA_389_) polymers bearing: 1) β-cyclodextrin (CD - the supramolecular host) (85 units/polymer); 2) Cy5 fluorophores for tracking the polymers (2 units/polymer); and 3) the TLR7 agonist-based adjuvant CL307 (57 units/polymer) (**Figure 1A**) – thus yielding the envisioned PbSPZ-SAS(CL307). Strong Cy5 signals could be observed along the surface of PbSPZ-SAS(CL307) indicative of effective chemical augmentation (**Figure 1B**). Flow cytometric analysis validated these findings and indicated about 90% of PbSPZ with SAS(CL307) present (**Figure 1C**; median fluorescent intensity of 27,965 for PbSPZ-SAS(CL307) versus 6 for control SPZ, p < 0.001).

### PbSPZ-SAS(CL307) induce a more pro-inflammatory response *in vitro* in macrophages

**Figure 2:**
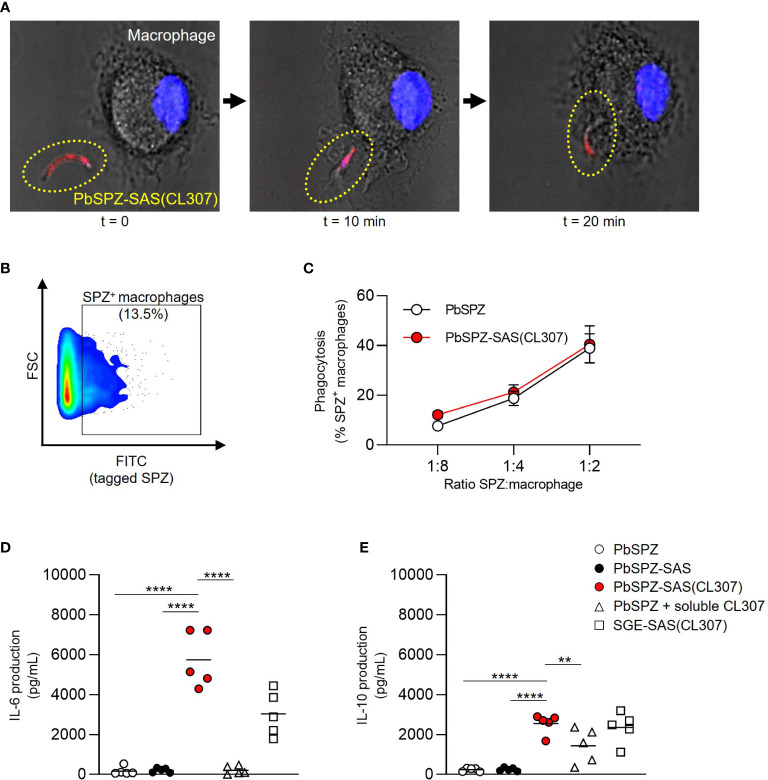
PbSPZ-SAS(CL307) induce a more pro-inflammatory response *in vitro* in macrophages. **(A)** Time-lapse confocal fluorescence microscopy images showing phagocytosis of a PbSPZ-SAS(CL307) (yellow dotted oval) by a bone marrow-derived macrophage (cell nucleus in blue). **(B)** Representative flow cytometry dot plot of macrophage cell size (y-axis) and FITC signal (x-axis) to isolate the proportion of macrophages (13.5%) positive for FITC-tagged PbSPZ. **(C)** The proportion of macrophages positive for FITC-tagged PbSPZ (y-axis) at differing ratios of SPZ:macrophages (x-axis) for PbSPZ-SAS(CL307) (red) versus control PbSPZ (white). Data of n = 3 from a representative experiment. **(D)** Production (pg/mL) of IL-6 cytokine (y-axis) by macrophages stimulated for 24h with PbSPZ-SAS(CL307) and controls. Data of n = 5 from three independent experiments. **(E)** Production (pg/mL) of IL-10 cytokine (y-axis) by macrophages stimulated for 24h with PbSPZ-SAS(CL307) and controls. Data of n = 5 biological replicates from three independent experiments. Statistical significance between groups was assessed by one-way ANOVA with multiple comparisons. PbSPZ, *P. berghei* sporozoite; SAS, supramolecular adjuvanting system; CL307, a Toll-like receptor 7 agonist; SGE, salivary gland extract; CD, cluster of differentiation; PD-L, programmed death ligand; IL, interleukin; **p < 0.01, ****p < 0.0001; figure legend: PbSPZ = wild-type PbSPZ (white circle), PbSPZ-SAS = PbSPZ with supramolecular polymer but lacking adjuvant (black circle), PbSPZ-SAS(CL307) = chemically adjuvanted PbSPZ (red circle), PbSPZ + soluble CL307 = wild-type PbSPZ + soluble adjuvant (white triangle), SGE-SAS(CL307) = chemically adjuvanted salivary gland extract (white square).

The immunological characteristics of PbSPZ-SAS(CL307) were first characterized *in vitro* using bone marrow-derived macrophages. Confocal imaging confirmed that PbSPZ-SAS(CL307) were normally phagocytosed by macrophages (**Figure 2A**). By fluorescently tagging PbSPZ with a FITC-based mitotracker we further assessed phagocytic uptake via flow cytometry (**Figure 2B**), and in so doing determined that the degree of phagocytosis of PbSPZ-SAS(CL307) was comparable to that of wild-type PbSPZ (**Figure 2C**; 21.3 ± 2.9% vs. 18.8 ± 2.9% at a ratio of 1 PbSPZ to 4 macrophages, p = 0.34).

Subsequently, we characterized the phenotype of macrophages after a 24-hour co-culture with PbSPZ-SAS(CL307). Overall, macrophage surface marker expression showed increased median expression of CD80 (**Figure S1A**; 3.1 ± 0.5 vs. 1.3 ± 0.3 fold-change over medium, p < 0.001) and PD-L1 (**Figure S2B**, 3.6 ± 0.3 vs. 1.8 ± 0.3 fold-change over medium, p < 0.001) for PbSPZ-SAS(CL307) compared to wild-type SPZ. Changes in surface marker expression were paralleled by large increases in cytokine production: IL-6 levels in response to PbSPZ-SAS(CL307) were increased 35-fold compared to wild-type PbSPZ (**Figure 2D**; 5,742 ± 1,391 vs. 166 ± 205 pg/mL, p < 0.0001), and increased 27-fold compared to both control PbSPZ-SAS (without adjuvant) (**Figure 2D**; 5,742 ± 1,391 vs. 211 ± 113 pg/mL, p < 0.0001) and to a cocktail of PbSPZ + concentration-matched soluble CL307 (**Figure 2D**; 5,742 ± 1,391 vs. 209 ± 206 pg/mL; p < 0.0001). Production of regulatory IL-10 was less dramatically increased, with 10-fold increases after incubation with PbSPZ-SAS(CL307) compared to both wild-type PbSPZ (**Figure 2E**; 2,543 ± 495 vs. 234 ± 89 pg/mL, p < 0.0001) and PbSPZ-SAS controls lacking adjuvant (**Figure 2E**; 2,543 ± 495 vs. 248 ± 89 pg/mL, p < 0.0001). However, compared to a PbSPZ + CL307 cocktail, IL-10 secretion by PbSPZ-SAS(CL307) was less than 2-fold increased (**Figure 2E**; 2,543 ± 495 vs. 1,431 ± 871 pg/mL, p < 0.01). Of note, comparable proportions of live cells between conditions (**Figure S3**) confirmed these results to be cytotoxicity-independent. Altogether, these data suggested that PbSPZ-SAS(CL307) induced immune responses *in vitro* that skewed distinctly more pro-inflammatory than those induced by wild-type PbSPZ – an effect notably superior to the addition of adjuvant in soluble form.

### Liver and spleen immune cells of mice immunized with PbSPZ-SAS(CL307) display a more proinflammatory phenotype after stimulation with PMA/ionomycin

**Figure 3:**
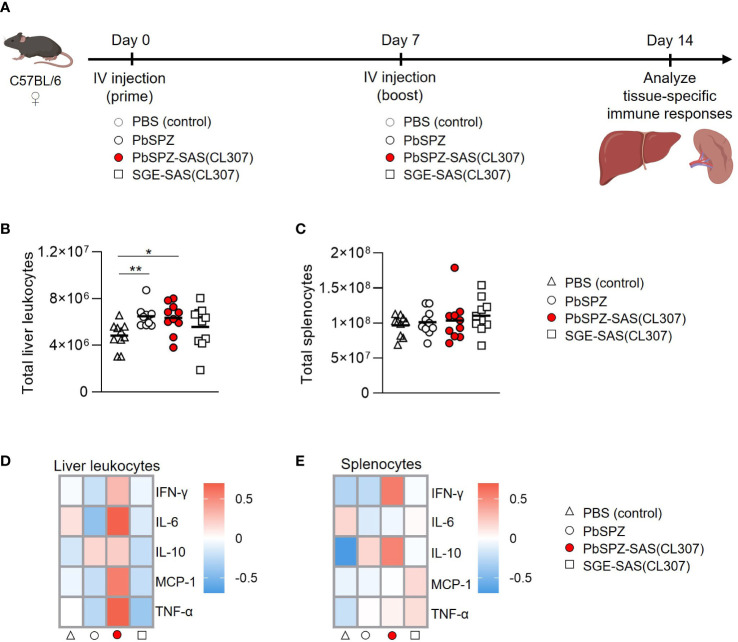
Liver and spleen immune cells of mice immunized with PbSPZ-SAS(CL307) display a more proinflammatory phenotype after stimulation with PMA/ionomycin. **(A)** Schematic of the experimental setup: mice were twice immunized at 1-week intervals with either PBS (white triangle), 25,000 PbSPZ (white circle), 25,000 PbSPZ-SAS(CL307) (red circle), or concentration-matched SGE-SAS(CL307) controls (white square). One week after the second immunization, livers and spleens were harvested and processed for immunophenotyping. **(B)** Total liver leukocyte counts (y-axis) of mice immunized with PbSPZ-SAS(CL307) or controls (x-axis). **(C)** Total splenocyte counts (y-axis) of mice immunized with PbSPZ-SAS(CL307) or controls (x-axis). **(D)** Heatmap of mean normalized values of secreted cytokines IFN-γ, IL-6, IL-10, MCP-1, and TNF-α (rows) by liver leukocytes from mice immunized with PbSPZ-SAS(CL307) or controls (columns) after 36 hours stimulation with PMA/ionomycin. **(E)** Heatmap of mean normalized values of secreted cytokines IFN-γ, IL-6, IL-10, MCP-1, and TNF-α (rows) by splenocytes from mice immunized with PbSPZ-SAS(CL307) or controls (columns) after 36 hours stimulation with PMA/ionomycin. All data shown are n = 10 biological replicates from two independent experiments. The statistical significance between groups was assessed by one-way ANOVA with multiple comparisons. PbSPZ, *P. berghei* sporozoites; SAS, supramolecular adjuvanting system; CL307, a Toll-like receptor 7 agonist; SGE, salivary gland extract; IFN, interferon; IL, interleukin; MCP, monocyte chemoattractant protein; TNF, tumor necrosis factor; PMA, phorbol 12-myristate 13-acetate; *p < 0.05.

To assess the *in vivo* immunogenicity of PbSPZ-SAS(CL307), female C57BL/6 mice were intravenously immunized twice at one-week intervals, followed by bulk immunophenotyping of liver and spleen at one week after the second immunization (**Figure 3A**). There was a 50% increase in number of liver leukocytes in mice immunized with PbSPZ irrespective of adjuvant status relative to PBS-immunized control mice (**Figure 3B**; 6.4 ± 1.3*10^6^ vs. 6.5 ± 0.9*10^6^ vs. 4.4 ± 1.1*10^6^ cells for PbSPZ-SAS(CL307) vs. PbSPZ vs. PBS, respectively, p < 0.05). In contrast to liver, splenocyte counts were mostly unaffected by PbSPZ-SAS(CL307) immunization, with comparable numbers found relative to controls immunized with PBS (**Figure 3C**; 1.03 ± 0.31*10^8^ vs. 9.7 ± 1.5*10^7^ cells, p = 0.54).

We next assessed the immune responsiveness of liver leukocytes and splenocytes towards a generic PMA/ionomycin stimulus in PbSPZ-SAS(CL307)-immunized animals as compared to controls. Liver leukocytes from PbSPZ-SAS(CL307)-immunized animals showed increased production of several pro-inflammatory cytokines (**Figure 3D**; **Figure S4**), most notably IL-6 (**Figure S5A**; mean fold-change of 0.67 vs. -0.42, p < 0.05) and TNF-α (**Figure S5A**; mean fold-change of 0.66 vs. -0.27, p < 0.05) compared to wild-type PbSPZ, though not in production of regulatory IL-10 (**Figure S5A**; mean fold-change of 0.21 vs. 0.17, p = 0.89). This effect appeared attributable to PbSPZ-SAS(CL307) themselves, as immunization with SGE-SAS(CL307) controls did not yield such increases (**Figure 3D**; **Figure S5A**). Splenocytes from mice immunized with PbSPZ-SAS(CL307) showed less evident increases in pro-inflammatory cytokine production (**Figure 3E**), limited to increased production of IFN-γ compared to wild-type PbSPZ (**Figure S5B**; mean fold-change of 0.56 vs. -0.25, p < 0.05). These data suggested that immunization with PbSPZ-SAS(CL307) induced more pro-inflammatory responsiveness, particularly in liver leukocytes.

### Liver leukocytes of PbSPZ-SAS(CL307)-immunized mice show enhanced SPZ-specific recall responses

**Figure 4:**
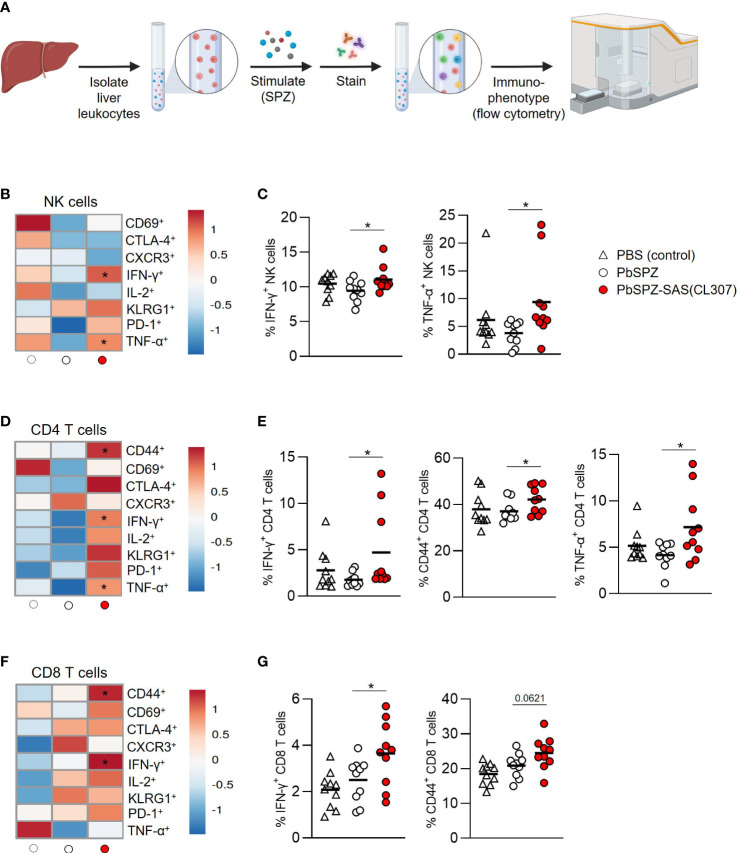
Liver leukocytes of PbSPZ-SAS(CL307)-immunized mice show enhanced SPZ-specific recall responses. **(A)** Schematic illustrating experimental setup. Harvested livers from immunized mice were processed to yield liver leukocytes that were stimulated with SPZ in the presence of BrefA, stained with a fluorescent antibody panel, and immunophenotyped by flow cytometry. **(B)** Heatmap of mean normalized values of activation markers CD69, CTLA-4, CXCR3, IFN-γ, IL-2, KLRG1, PD-1, and TNF-α in NK cells (rows) after SPZ stimulation of liver leukocytes of mice immunized with PbSPZ-SAS(CL307) or controls (columns). **(C)** Frequency of IFN-γ^+^ and TNF-α^+^ NK cells (y-axis) after SPZ stimulation of liver leukocytes of mice immunized PbSPZ-SAS(CL307) or controls (x-axis). **(D)** Heatmap of mean normalized values of activation markers CD44, CD69, CTLA-4, CXCR3, IFN-γ, IL-2, KLRG1, PD-1, and TNF-α in CD4^+^ T cells (rows) after SPZ stimulation of liver leukocytes of mice immunized with PbSPZ-SAS(CL307) hybrids or controls (columns). **(E)** Frequency of IFN-γ^+^, CD44^+^, and TNF-α^+^ CD4^+^ T cells (y-axis) after SPZ stimulation of liver leukocytes of mice immunized with PbSPZ-SAS(CL307) or controls (x-axis). **(F)** Heatmap of mean normalized values of activation markers CD44, CD69, CTLA-4, CXCR3, IFN-γ, IL-2, KLRG1, PD-1, and TNF-α in CD8^+^ T cells (rows) after SPZ/BrefA stimulation of liver leukocytes of mice immunized with PbSPZ-SAS(CL307) or controls (columns). **(G)** Frequency of IFN-γ^+^, CD44^+^, and TNF-α^+^ CD8^+^ T cells (y-axis) after SPZ stimulation of liver leukocytes of mice immunized PbSPZ-SAS(CL307) or controls (x-axis). All data shown are n = 10 biological replicates from two independent experiments. Statistical significance between groups was assessed by one-way ANOVA with multiple comparisons. PbSPZ, *P. berghei* sporozoite; SAS, supramolecular adjuvanting system; CL307, a Toll-like receptor 7 agonist; CD, cluster of differentiation, CTLA, cytotoxic T-lymphocyte-associated protein; CXCR, chemokine receptor; IFN, interferon; IL, interleukin; KLRG, killer cell lectin-like receptor; PD, programmed death; TNF, tumor necrosis factor; BrefA = brefeldin A; *p < 0.05; figure legend: PBS = vehicle (negative control – white triangle), PbSPZ = wild-type SPZ (white circle), PbSPZ-SAS(CL307) = chemically adjuvanted SPZ (red circle).

Given the overall increased pro-inflammatory responsiveness of liver leukocytes after PbSPZ-SAS(CL307) immunization, we next evaluated SPZ-specific activation of liver leukocytes by co-culturing them with SPZ for 4 hours, followed by flow cytometric immunophenotyping (**Figure 4A**). Here, we found increased frequencies of activated cells in several different compartments for PbSPZ-SAS(CL307)-immunized animals. In the myeloid compartment (defined as CD45^+^CD3^-^B220^-^ cells; see **Figures S6, S7A**), we observed an increased frequency of activated CD86^+^ cells in PbSPZ-SAS(CL307)-immunized mice compared to wild-type PbSPZ (**Figure S7B**, 25.4 ± 4.3 vs. 21.3 ± 2.5%, p < 0.05), as well as increased frequencies in CD11b^+^ (**Figure S7B**, 23.3 ± 4.3 vs. 18.8 ± 3.9%, p < 0.05) and CD11c^+^ (**Figure S7B**, 26.7 ± 4.6 vs. 20.6 ± 3.0%, p < 0.01) subsets. Liver NK cells (**Figure S8**) of mice immunized with PbSPZ-SAS(CL307) also showed increased expression of several activation markers compared to those immunized with wild-type PbSPZ (**Figure 4B**), including significant increases in IFN-γ^+^ (**Figure 4C**; 11.1 ± 1.8 vs. 9.4 ± 1.5% IFN-γ^+^ NK cells, p < 0.05) and TNF-α^+^ (**Figure 4C**; 9.4 ± 7.2 vs. 3.8 ± 2.1% TNF-α^+^ NK cells, p < 0.05) NK cells. Similarly, increased activation in PbSPZ-SAS(CL307)-immunized mice was furthermore seen for CD4^+^ T cells (**Figure 4D**; **Figure S8**) and CD8^+^ T cells (**Figure 4F**; **Figure S8**). In particular, CD4^+^ T cells showed significantly increased frequencies of IFN-γ^+^ (**Figure 4E**; 4.7 ± 4.3 vs. 1.8 ± 0.7% IFN-γ^+^ CD4 T cells, p < 0.05), CD44^+^ (**Figure 4E**; 42.1 ± 5.8 vs. 37.1 ± 4.3% CD44^+^ CD4 T cells, p < 0.05), and TNF-α^+^ (**Figure 4E**; 7.2 ± 3.7 vs. 4.2 ± 1.3% TNF-α^+^ CD4 T cells, p < 0.05) cells; CD8^+^ T cells showed significant increases in frequency of IFN-γ^+^ (**Figure 4G**; 3.6 ± 1.4 vs. 2.5 ± 0.9% IFN-γ^+^ CD8 T cells, p < 0.05) and increased frequency in CD44^+^ (**Figure 4G**; 24.5 ± 4.6 vs. 20.9 ± 3.4% CD44^+^ CD8 T cells, p = 0.0621) cells. Notably, there was considerable within-group heterogeneity of SPZ-specific activation, with some mice showing exceptionally high cytokine profiles and others more comparable to controls, though the effects were nevertheless consistent between experiments (**Figure S9**). Altogether, these results indicated an enhanced SPZ-specific pro-inflammatory response of important subsets of both innate and adaptive immune cells in the livers of mice immunized with PbSPZ-SAS(CL307).

## Discussion

Here, we have shown the conceptual feasibility of chemically augmenting SPZ with adjuvants to alter their immunogenicity both *in vitro* and *in vivo*. Macrophages stimulated *in vitro* with chemically augmented PbSPZ-SAS(CL307) displayed a more pro-inflammatory phenotype, especially with regard to increased production of IL-6. *In vivo*, intravenous immunization of mice with PbSPZ-SAS(CL307) skewed in particular liver leukocytes towards more pro-inflammatory responses in response to a generic PMA/ionomycin stimulus, and furthermore induced an enhanced pro-inflammatory, SPZ-specific activation of both innate (e.g. CD86^+^ myeloid; IFN-γ^+^ NK cells) and adaptive (e.g. IFN-γ^+^ CD4^+^ and CD8^+^ T cells) liver cell compartments.

There exists a clear need for innovative strategies that increase the potency, i.e. immunogenicity, of SPZ-based vaccines. Our findings extend the work that has previously established the feasibility of improving SPZ immunogenicity via the traditional adjuvanting method of administering a mixture of SPZ and adjuvant (27–30). Specifically, we found that chemically augmented SPZ-SAS(CL307) can be stably formed and show considerably boosted immunogenicity *in vitro*, with 35-fold increases in IL-6 production by macrophages compared to unadulterated SPZ. This large increase in immunogenicity seemed, in particular, attributable to the physical co-localization ensured by a supramolecularly complexed adjuvant, as SPZ-SAS(CL307) induced 27-fold increases in IL-6 production compared to a mixture of SPZ and CL307. We thus conclude that chemical augmentation may provide a platform for realizing vaccine/adjuvant co-localization with whole SPZ-based vaccines, which could feasibly address the challenge of achieving targeted immune activation with the intravenous administration of such vaccines. Importantly, preliminary studies in our group show that such chemical augmentation is also feasible with human *P. falciparum* parasites, indicating the potential for translating this approach to humans.

In this proof-of-concept study for chemically augmenting SPZ immunogenicity, we chose a TLR7 agonist as the adjuvanting moiety, given their excellent track record of inducing cytotoxic T-cell responses (23–25). Such responses, in particular, manifested in the form of IFN-γ^+^ immune cells and are thought to be important mediators of malarial protection (5–7), making a TLR7 agonist a logical first choice. We found that chemical augmentation of SPZ with a TLR7 agonist did indeed induce more cytotoxic responses in immunized mice’s livers, with significantly increased frequencies of IFN-γ^+^ NK cells, CD4^+^ T cells, and CD8^+^ T cells upon SPZ restimulation. These findings suggest that 1) physically co-localized adjuvants retain their immunogenic properties for eliciting specific types of immune responses, and 2) that these immune responses are coupled to the antigenic stimulus – in this case, SPZ. Future modifications of this proof-of-concept would thus be well-suited for producing refinements of PbSPZ-SAS(CL307) that, for example, harness the synergistic effects reported between different TLR agonists (31, 32). Such synergistic effects have been shown capable of boosting the expression of pro-inflammatory markers without the concomitant increase in regulatory signaling, such as the PDL-1/PD-1 axis, that typically results from immunogen stimulation (33), which could be a key aspect in ensuring that activation is not impeded by increased signaling along such regulatory axes.

The proof-of-concept shown here does not take into account the potential role of SPZ motility in vaccine efficacy. In adapting our SAS technology to SPZ, we found that motile SPZ mostly shed surface-localized adjuvant – an effect similarly described in antibody studies (34). Robust chemical augmentation thus necessitated the immobilization of SPZ. The use of immobilized SPZ for vaccination does present certain advantages, especially in obviating the need for costly liquid nitrogen storage and transportation logistics, but comes at the cost of decreasing protective efficacy by preventing the infection of hepatocytes that seem to most optimally stimulate an immune response (35). Hence, subsequent refinements of the concept would do well to explore the feasibility of anchoring adjuvant intracellularly as a means of preserving SPZ motility and infectivity.

In conclusion, we have here provided a proof-of-concept indicating the potential for chemically altering the immunogenic profile of malaria SPZ. Further refinements of the concept could pave the way toward generating more efficacious SPZ-based vaccines for use in malaria-endemic areas.

## Data availability statement

The raw data supporting the conclusions of this article will be made available by the authors, without undue reservation.

## Ethics statement

The animal study was reviewed and approved by LUMC PDC.

## Author contributions

Conceptualization, ND, FL, and MR; Methodology, ND, BF-F, FL, and MR; Investigation, ND, RS, SC-M, LB-R, SW, CN, GH, and AB; Resources, DW and EB; Writing – Original Draft, ND, FL and MR; Writing – Review and Editing, ND, FL, and MR; Supervision, AV, BF-F, FL, and MR; Funding Acquisition, FL and MR. All authors contributed to the article and approved the submitted version.
