# Supplementary material for: Chemically augmented malaria sporozoites display an altered immunogenic profile
Source: Front Immunol. 2023 Aug 31;14:1204606. doi: 10.3389/fimmu.2023.1204606 (PMC10500441; doi:10.3389/fimmu.2023.1204606)
Supplement: Supplementary file 1 [file DataSheet_1.docx]

Supplementary Material

Chemically augmented malaria sporozoites display an altered immunogenic profile

**Supplementary figures**

**
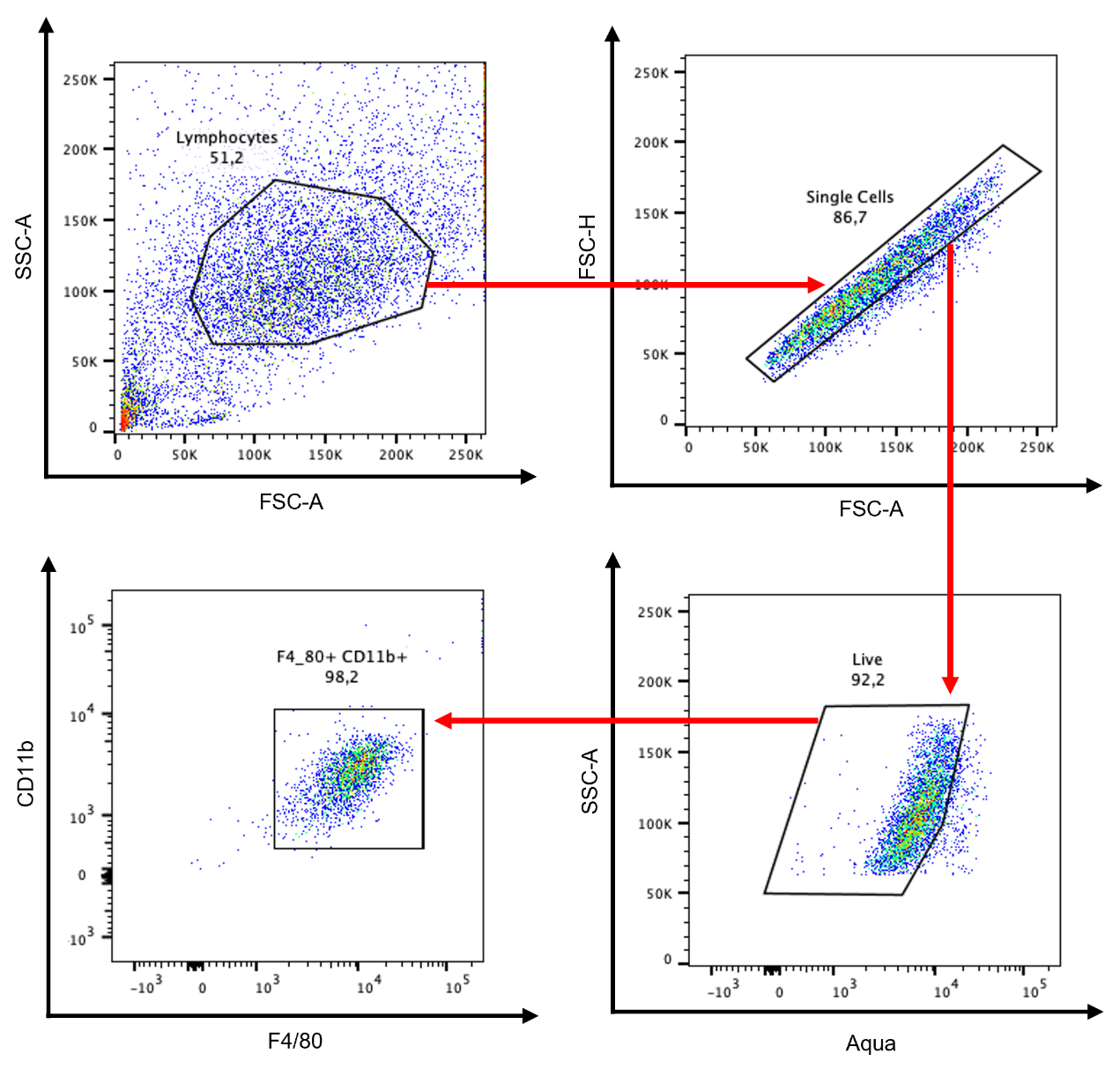
**

**Supplementary Figure 1: Gating strategy to isolate macrophage population for expression marker analysis post stimulation with PbSPZ-SAS(CL307) and controls.**

**
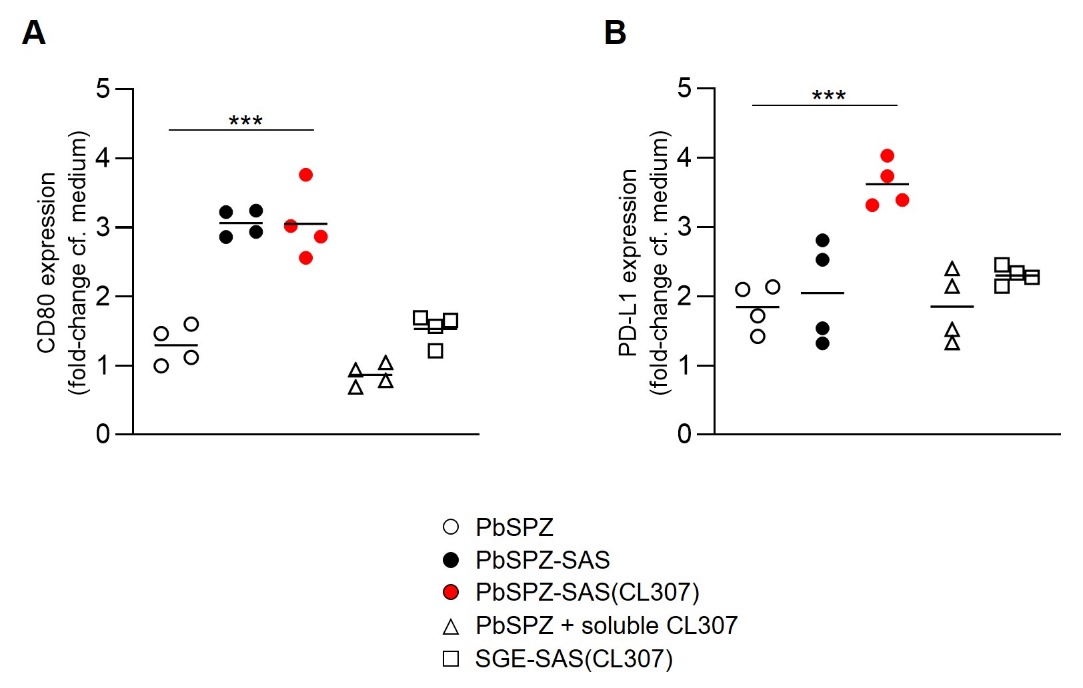
**

**Supplementary Figure 2: PbSPZ-SAS(CL307) induce a more pro-inflammatory response *in vitro* in macrophages. (A)** Fold-change (relative to medium) of surface marker CD80 (y-axis) in macrophages stimulated for 24h with PbSPZ-SAS(CL307) and controls. **(B)** Fold-change (relative to medium) of surface marker PD-L1 (y-axis) in macrophages stimulated for 24h with PbSPZ-SAS(CL307) and controls. Data shown are n = 4 biological replicates from three independent experiments. Statistical significance between groups was assessed by one-way ANOVA with multiple comparisons. PbSPZ = *P. berghei* sporozoite; SGE = salivary gland extract; SAS = supramolecular adjuvanting system; CL307 = a Toll-like receptor 7 agonist; CD = cluster of differentation; *** = p < 0.001; figure legend: PbSPZ = wild-type PbSPZ (white circle), PbSPZ-SAS = PbSPZ with supramolecular polymer but lacking adjuvant (black circle), PbSPZ-SAS(CL307) = chemically adjuvanted PbSPZ (red circle), PbSPZ + soluble CL307 = wild-type PbSPZ + soluble adjuvant (white triangle), SGE-SAS(CL307) = chemically adjuvanted salivary gland extract (white square).

**
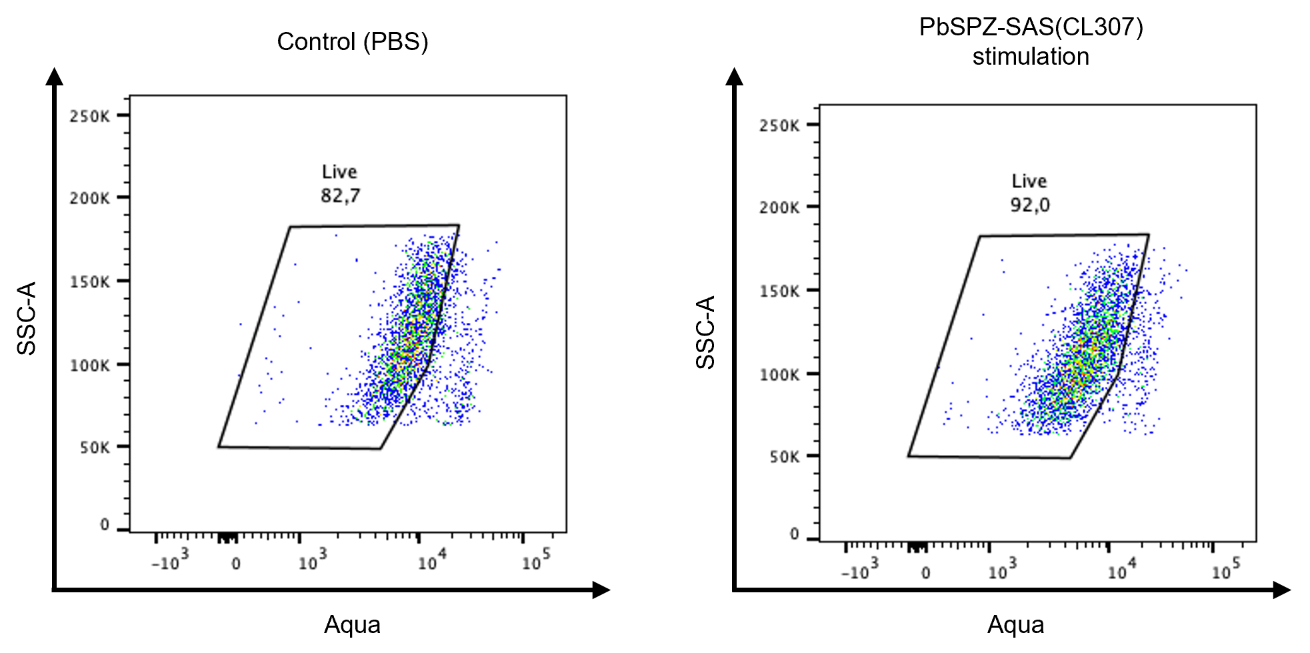
**

**Supplementary Figure 3: Representative plots of proportion live cells after *in vitro* stimulation of macrophages with PBS (control, left) versus PbSPZ-SAS(CL307).**

S(CL307) = chemically adjuvanted SGE (black square).


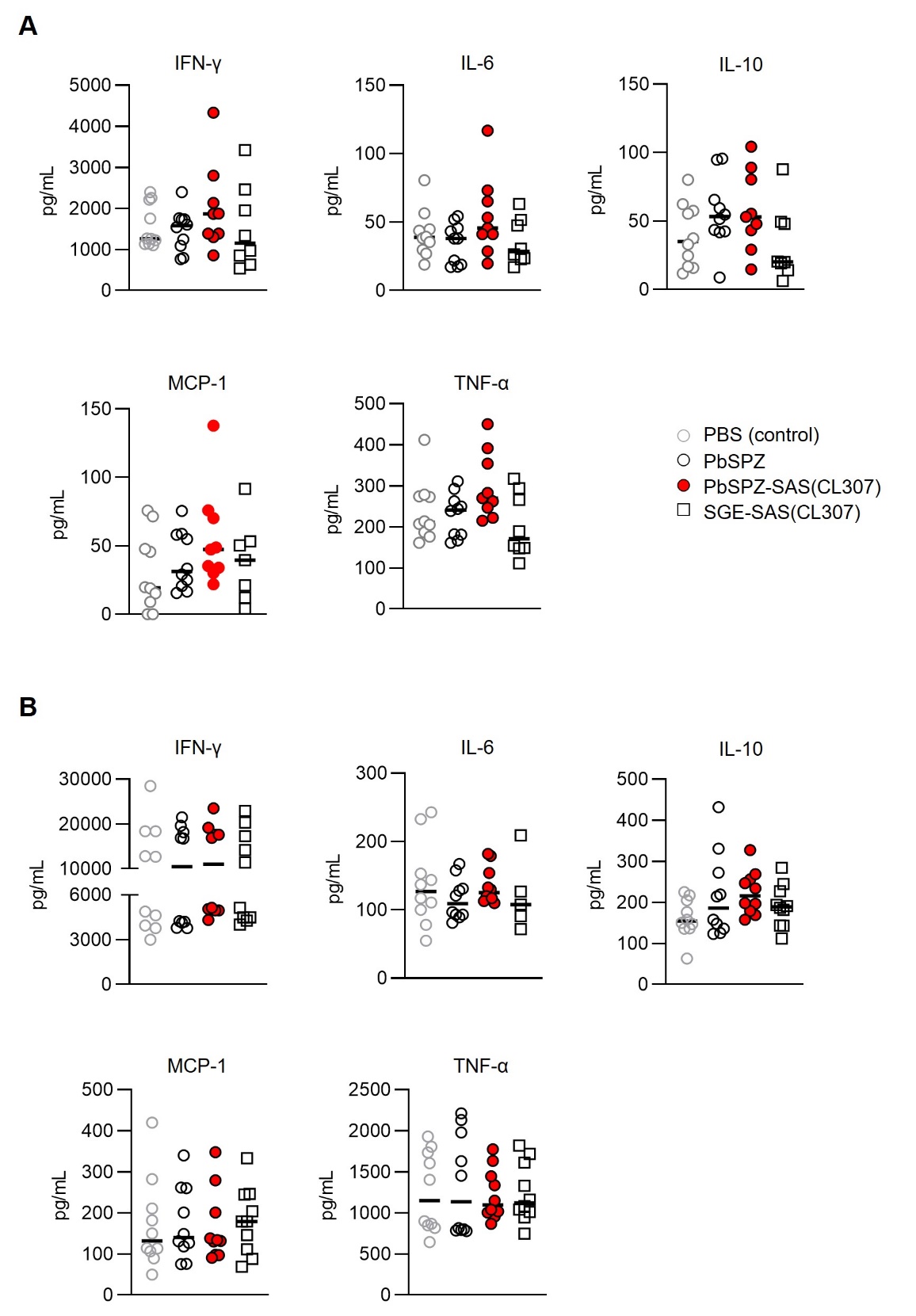


**Supplementary Figure 4: Liver and spleen immune cells of mice immunized with PbSPZ-SAS(CL307) tend to secrete more proinflammatory cytokines after stimulation with PMA/ionomycin. (A)** Levels (pg/mL) of secreted cytokines IFN-γ, IL-6, IL-10, MCP-1 and TNF-α (y-axis) by liver leukocytes from mice immunized with PbSPZ-SAS(CL307) or controls (columns) after 36 hours stimulation with PMA/ionomycin. **(B)** Levels (pg/mL) of secreted cytokines IFN-γ, IL-6, IL-10, MCP-1 and TNF-α (y-axis) by splenocytes from mice immunized with PbSPZ-SAS(CL307) or controls (columns) after 36 hours stimulation with PMA/ionomycin. Data shown are n = 10 biological replicates from two independent experiments. PbSPZ = *P. berghei* sporozoite; SGE = salivary gland extract; SAS = supramolecular adjuvanting system; CL307 = a Toll-like receptor 7 agonist; CD = cluster of differentation; figure legend: PBS = vehicle (negative control – grey circle), PbSPZ = wild-type SPZ (black circle), PbSPZ-SAS(CL307) = chemically adjuvanted SPZ (red circle), SGE-SAS(CL307) = chemically adjuvanted SGE (black square).


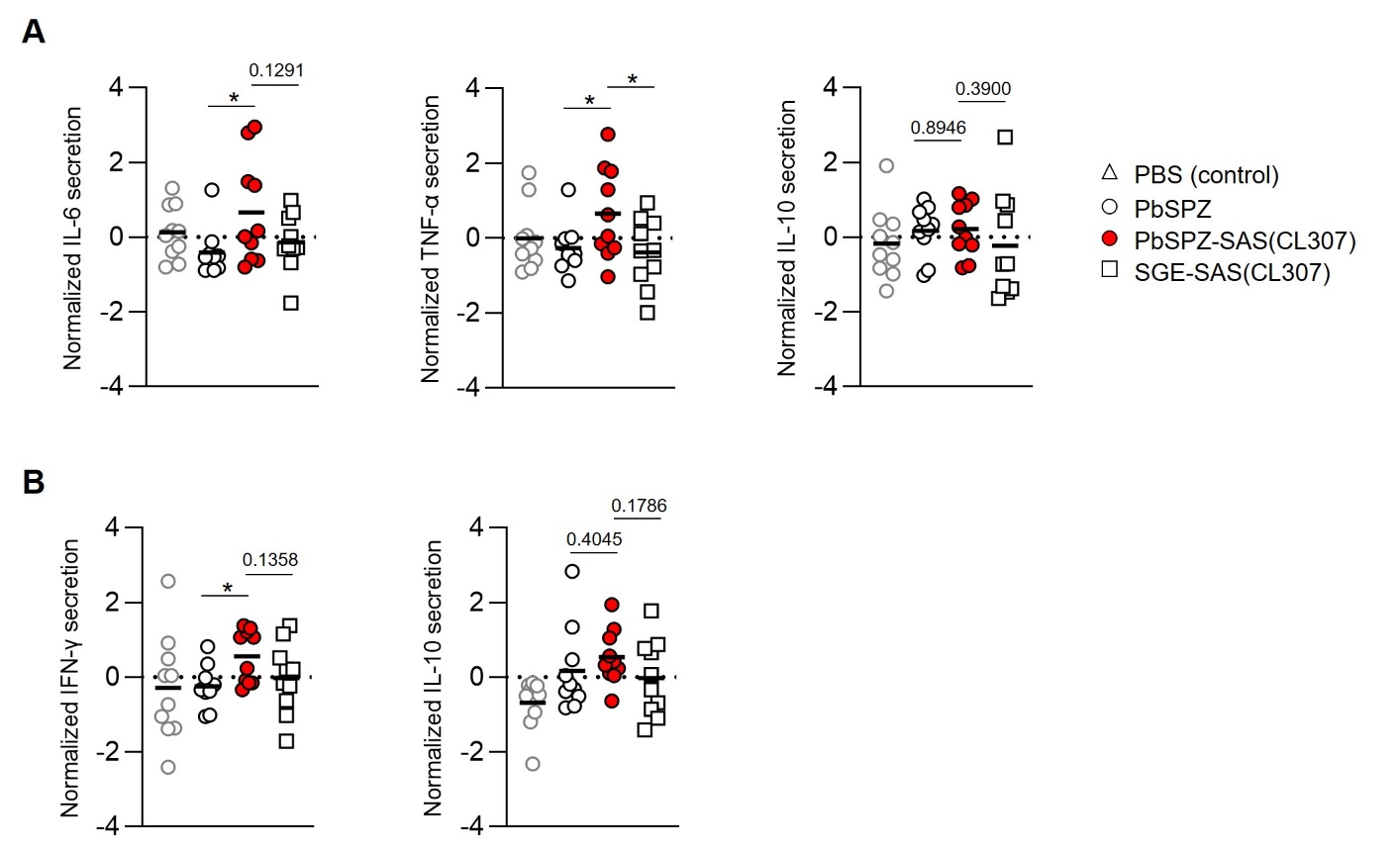


**Supplementary Figure 5: Liver and spleen immune cells of mice immunized with PbSPZ-SAS(CL307) display a more proinflammatory phenotype after stimulation with PMA/ionomycin. (A)** Normalized values of secreted cytokines IL-6, TNF-α and IL-10 (y-axis) by liver leukocytes from mice immunized with PbSPZ-SAS(CL307) or controls (columns) after 36 hours stimulation with PMA/ionomycin. **(B)** Normalized values of secreted cytokines IFN-γ and IL-10 (y-axis) by splenocytes from mice immunized with PbSPZ-SAS(CL307) or controls (columns) after 36 hours stimulation with PMA/ionomycin. Data shown are n = 10 biological replicates from two independent experiments. Statistical significance between groups was assessed by one-way ANOVA with multiple comparisons. PbSPZ = *P. berghei* sporozoite; SGE = salivary gland extract; SAS = supramolecular adjuvanting system; CL307 = a Toll-like receptor 7 agonist; CD = cluster of differentation; * = p < 0.05; figure legend: PBS = vehicle (negative control – grey circle), PbSPZ = wild-type SPZ (black circle), PbSPZ-SAS(CL307) = chemically adjuvanted SPZ (red circle), SGE-SA


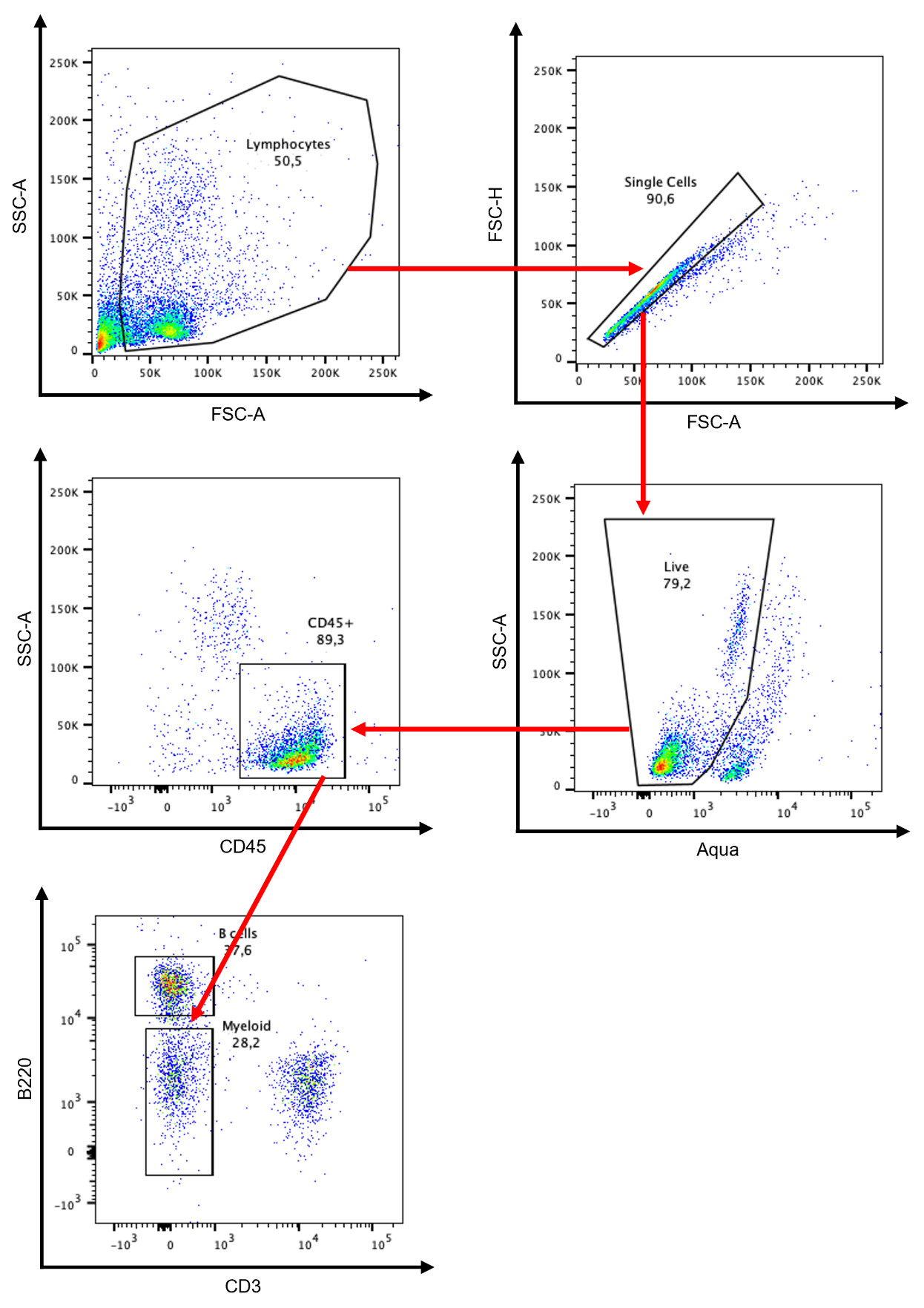


**Supplementary Figure 6: Gating strategy to isolate myeloid cells from livers of mice immunized with PbSPZ-SAS(CL307) and controls for expression marker analysis by flow cytometry.**


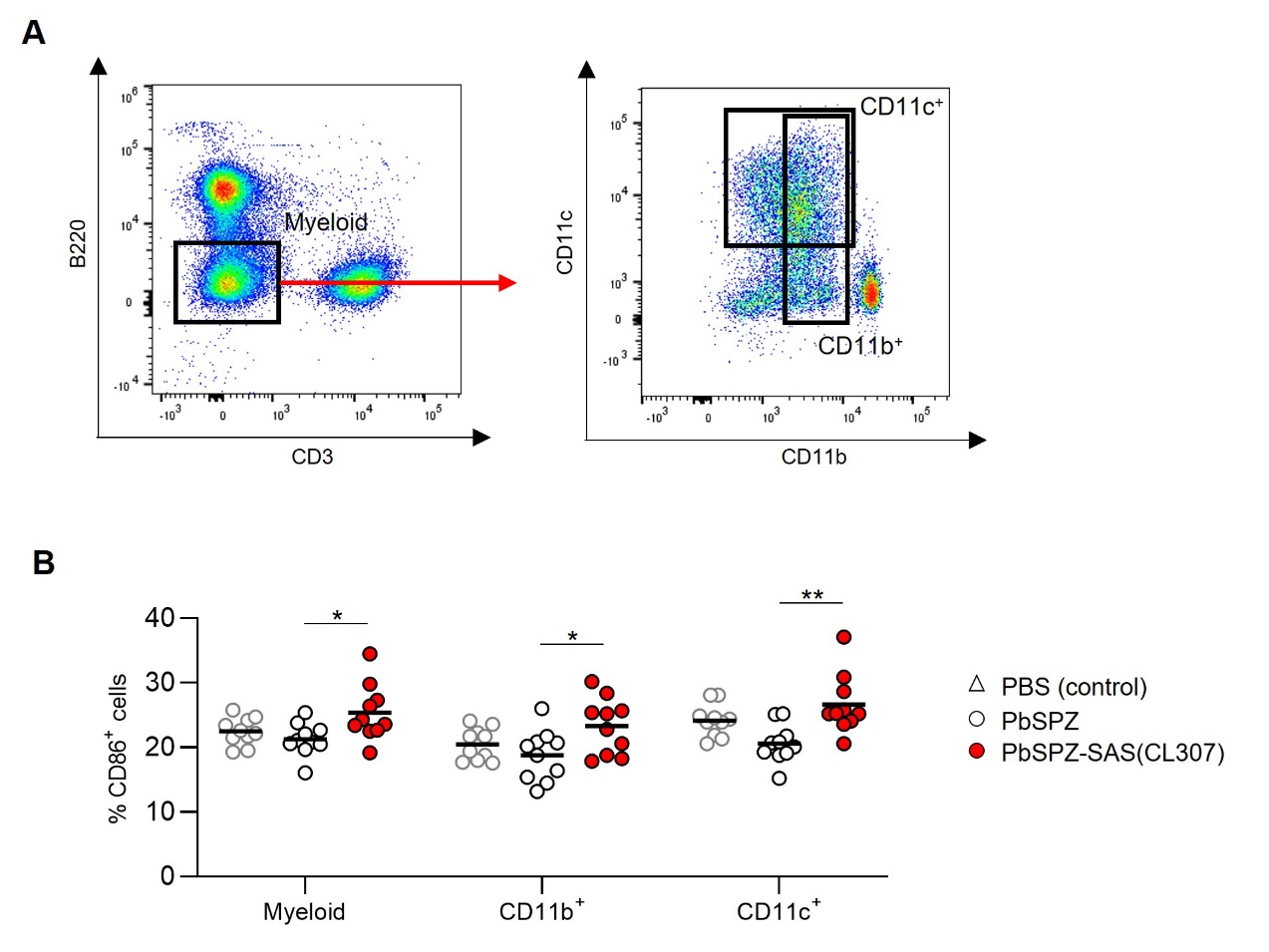


**Supplementary Figure 7: Myeloid liver leukocytes of PbSPZ-SAS(CL307)-immunized mice show enhanced SPZ-specific recall responses. (A)** Gating strategy for isolating myeloid liver cells (defined as CD45^+^ B220^-^ CD3^-^ cells), as well as CD11b^+^ and CD11c^+^ subpopulations therein. **(B)** Frequency activated CD86^+^ cells (y-axis) in myeloid, CD11b^+^ and CD11c^+^ cell compartments (x-axis) in livers of mice immunized with PBS (white triangle), control PbSPZ (white circle) and chemically augmented PbSPZ-SAS(CL307) (red circle). Data shown are n = 10 biological replicates from two independent experiments. Statistical significance between groups was assessed by one-way ANOVA with multiple comparisons. PbSPZ = *P. berghei* sporozoite; SAS = supramolecular adjuvanting system; CL307 = a Toll-like receptor 7 agonist; CD = cluster of differentation; * = p < 0.05, ** = p < 0.01; figure legend: PBS = vehicle (negative control – white triangle), PbSPZ = wild-type SPZ (white circle), PbSPZ-SAS(CL307) = chemically adjuvanted SPZ (red circle).


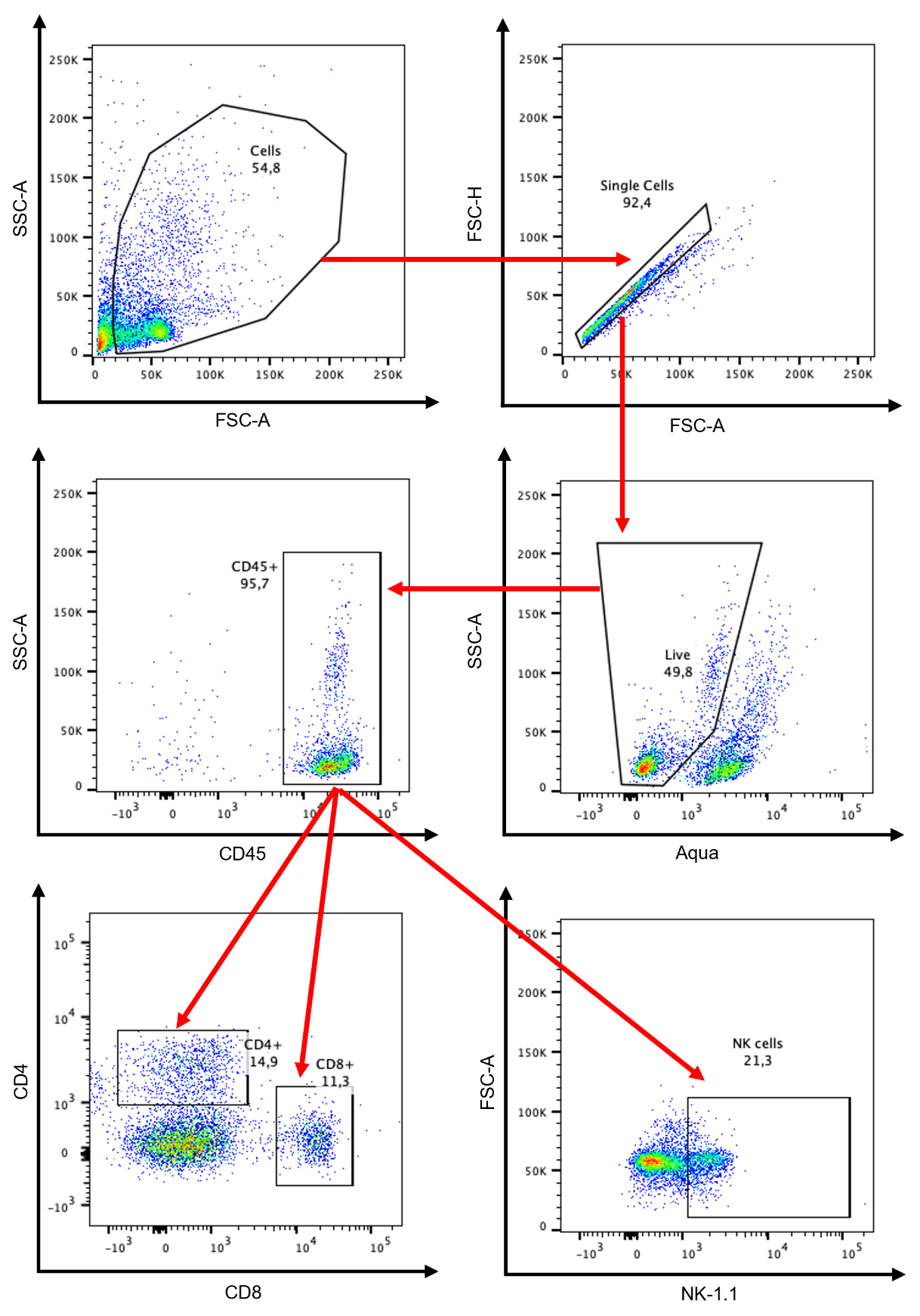


**Supplementary Figure 8: Gating strategy to isolate CD4 T cells, CD8 T cells and NK cells from livers of mice immunized with PbSPZ-SAS(CL307) and controls for expression marker analysis by flow cytometry.**

**
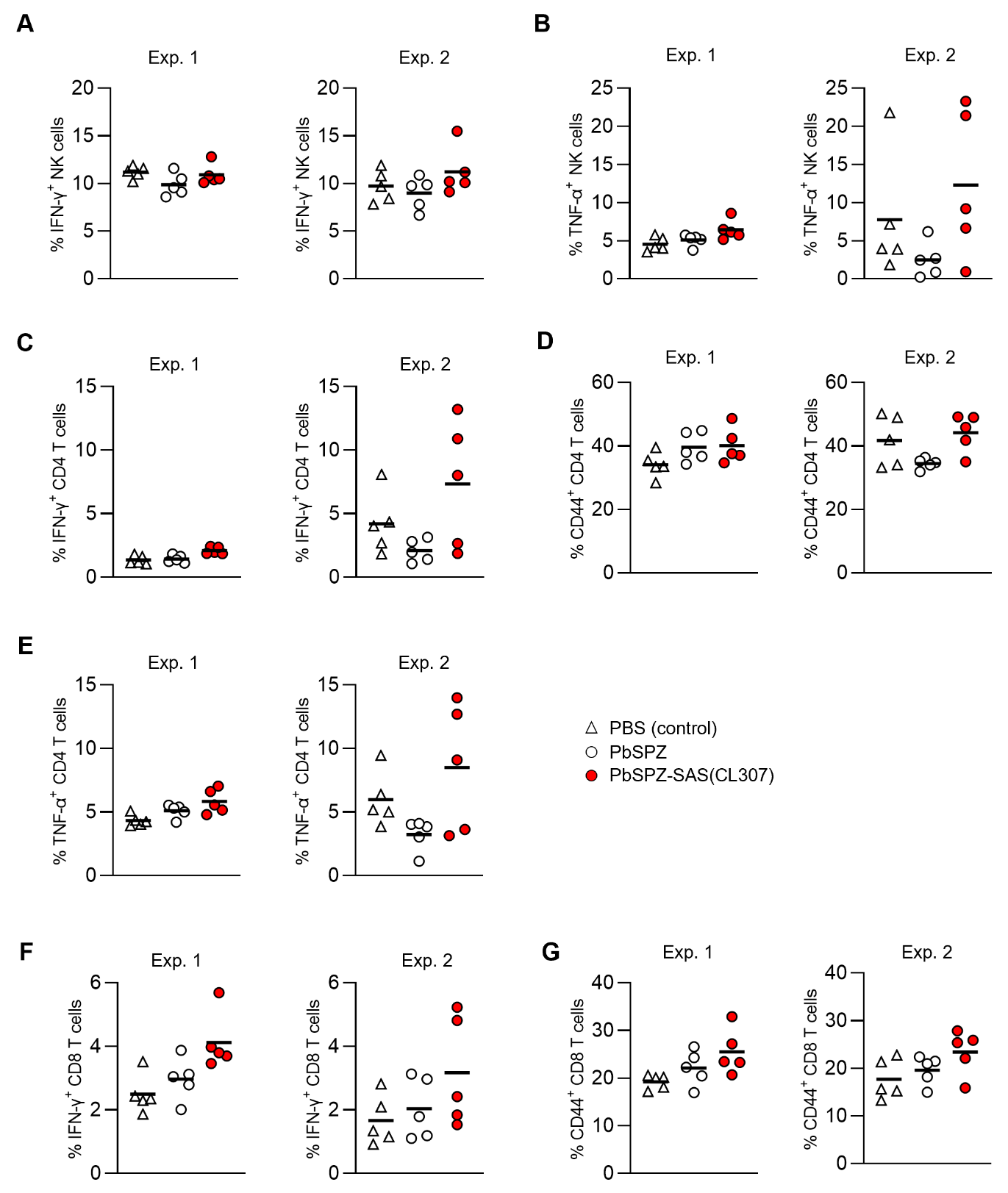
**

**Supplementary Figure 9: Consistent increases in pro-inflammatory markers of NK cells, CD4 T cells and CD8 T cells in response to PbSPZ-SAS(CL307) immunization was found between two independent experiments. (A)** Frequency of IFN-γ^+^ NK cells (y-axis) after SPZ stimulation of liver leukocytes of mice immunized PbSPZ-SAS(CL307) or controls (x-axis) between experiments #1 (left) and experiments #2 (right). **(B)** Frequency of TNF-α^+^ NK cells (y-axis) after SPZ stimulation of liver leukocytes of mice immunized PbSPZ-SAS(CL307) or controls (x-axis) between experiments #1 (left) and experiments #2 (right). **(C)** Frequency of IFN-γ^+^ CD4 T cells (y-axis) after SPZ stimulation of liver leukocytes of mice immunized PbSPZ-SAS(CL307) or controls (x-axis) between experiments #1 (left) and experiments #2 (right). **(D)** Frequency of CD44^+^ CD4 T cells (y-axis) after SPZ stimulation of liver leukocytes of mice immunized PbSPZ-SAS(CL307) or controls (x-axis) between experiments #1 (left) and experiments #2 (right). **(E)** Frequency of TNF-α^+^ CD4 T cells (y-axis) after SPZ stimulation of liver leukocytes of mice immunized PbSPZ-SAS(CL307) or controls (x-axis) between experiments #1 (left) and experiments #2 (right). **(F)** Frequency of IFN-γ^+^ CD8 T cells (y-axis) after SPZ stimulation of liver leukocytes of mice immunized PbSPZ-SAS(CL307) or controls (x-axis) between experiments #1 (left) and experiments #2 (right). **(G)** Frequency of CD44^+^ CD8 T cells (y-axis) after SPZ stimulation of liver leukocytes of mice immunized PbSPZ-SAS(CL307) or controls (x-axis) between experiments #1 (left) and experiments #2 (right). PbSPZ = *P. berghei* sporozoite; SAS = supramolecular adjuvanting system; CL307 = a Toll-like receptor 7 agonist; CD = cluster of differentiation, TNF = tumor necrosis factor; figure legend: PBS = vehicle (negative control – white triangle), PbSPZ = wild-type SPZ (white circle), PbSPZ-SAS(CL307) = chemically adjuvanted SPZ (red circle).


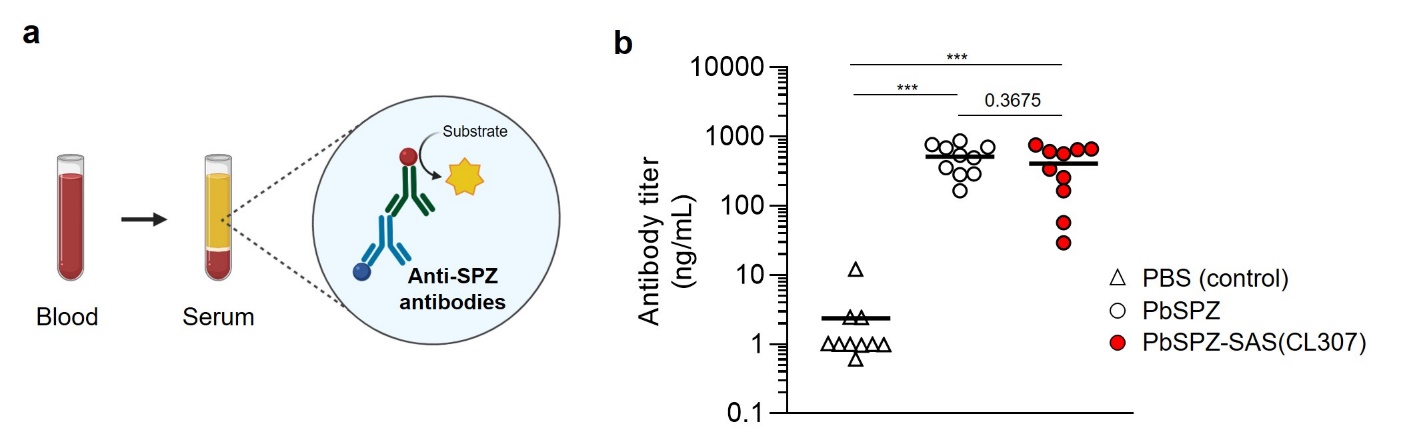


**Supplementary Figure 10: PbSPZ-SAS(CL307)-immunized mice do not show increased anti-SPZ antibody levels. (A)** Schematic of experimental setup: blood harvested from immunized mice was immediately spun down to yield serum assayed for presence of anti-SPZ antibodies by means of an in-house enzyme-linked immunosorbent assay. **(B)** Titers of anti-SPZ antibodies (ng/mL, y axis) in blood of mice immunized with PBS (white triangle), control PbSPZ (white circle) and chemically augmented PbSPZ-SAS(CL307) (red circle). Data shown are n = 10 biological replicates from two independent experiments. Statistical significance between groups was assessed by one-way ANOVA with multiple comparisons. PbSPZ = *P. berghei* sporozoite; SAS = supramolecular adjuvanting system; CL307 = a Toll-like receptor 7 agonist; *** = p < 0.001; figure legend: PBS = vehicle (negative control – white triangle), PbSPZ = wild-type SPZ (white circle), PbSPZ-SAS(CL307) = chemically adjuvanted SPZ (red circle).


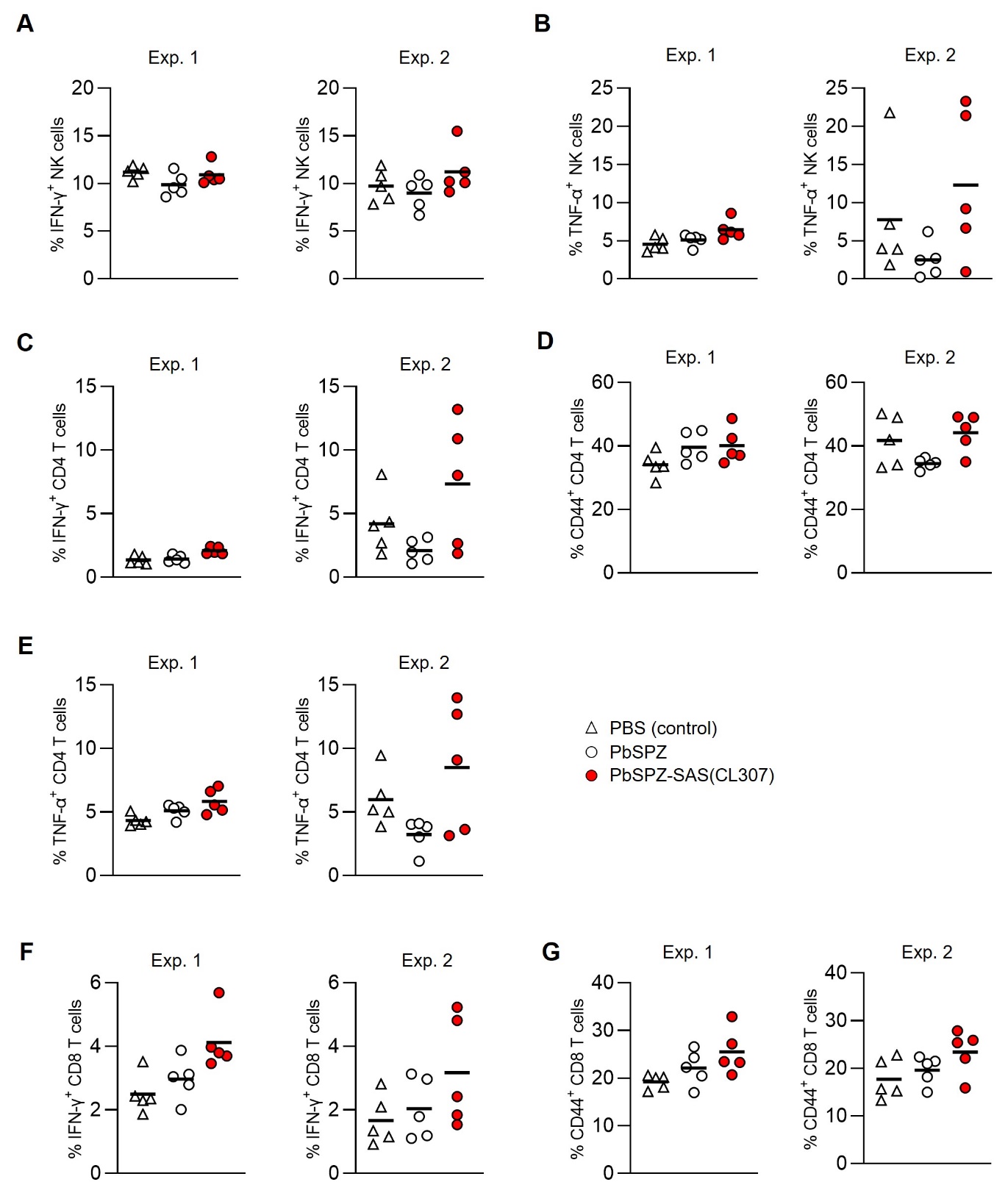


**Supplementary Figure 11: Enhanced SPZ-specific recall responses by liver leukocytes of mice immunized with PbSPZ-SAS(CL307) were consistently found between experiments. (A)** Frequency of IFN-γ^+^ NK cells (y-axis) after SPZ stimulation of liver leukocytes of mice immunized with PbSPZ-SAS(CL307) or controls (x-axis). **(B)** Frequency of TNF-α^+^ NK cells (y-axis) after SPZ stimulation of liver leukocytes of mice immunized with PbSPZ-SAS(CL307) or controls (x-axis). **(C)** Frequency of IFN-γ^+^ CD4^+^ T cells (y-axis) after SPZ stimulation of liver leukocytes of mice immunized with PbSPZ-SAS(CL307) or controls (x-axis). **(D)** Frequency of CD44^+^ CD4^+^ T cells (y-axis) after SPZ stimulation of liver leukocytes of mice immunized with PbSPZ-SAS(CL307) or controls (x-axis). **(E)** Frequency of TNF-α^+^ CD4^+^ T cells (y-axis) after SPZ stimulation of liver leukocytes of mice immunized with PbSPZ-SAS(CL307) or controls (x-axis). **(F)** Frequency of IFN-γ^+^ CD8^+^ T cells (y-axis) after SPZ stimulation of liver leukocytes of mice immunized with PbSPZ-SAS(CL307) or controls (x-axis). **(G)** Frequency of CD44^+^ CD8^+^ T cells (y-axis) after SPZ stimulation of liver leukocytes of mice immunized with PbSPZ-SAS(CL307) or controls (x-axis). PbSPZ = *P. berghei* sporozoite; SAS = supramolecular adjuvanting system; CL307 = a Toll-like receptor 7 agonist; CD = cluster of differentiation, IFN = interferon, TNF = tumor necrosis factor; figure legend: PBS = vehicle (negative control – white triangle), PbSPZ = wild-type SPZ (white circle), PbSPZ-SAS(CL307) = chemically adjuvanted SPZ (red circle).
